# Anti-Thrombotic and Metabolic Protective Effects of Ginseng in High-Fat Diet-Induced Obese Rats: In Vivo Evaluation with In Silico Mechanistic Prediction

**DOI:** 10.3390/antiox15080931

**Published:** 2026-07-27

**Authors:** Eun-Jin Lee, Dahye Yoon, Woo-Cheol Shin, Bo-Ram Choi, Dash Oyunbileg, Hye Yoon Do, Sun-Seek Min, Jin Seong Kim, Dae Young Lee, Dae-Yong Song

**Affiliations:** 1 Department of Anatomy and Neuroscience, Eulji University School of Medicine, Daejeon 34824, Republic of Korea; hutcom08@naver.com (E.-J.L.);; 2 Department of Herbal Crop Research, National Institute of Horticultural and Herbal Science, Rural Development Administration, Eumseong 27709, Republic of Korea; dahyeyoon@korea.kr (D.Y.);; 3 BK21 FOUR KNU Creative BioResearch Group, School of Life Sciences, Kyungpook National University, Daegu 41566, Republic of Korea; suc0094@knu.ac.kr; 4 Department of Physiology and Biophysics, Eulji University School of Medicine, Daejeon 34824, Republic of Korea; 5 Geumsan Ginseng and Herb Development Agency, Geumsan 32724, Republic of Korea

**Keywords:** white ginseng extract, anti-thrombotic effect, cardiovascular disease, ginsenosides, in silico analysis

## Abstract

Cardiovascular disease (CVD) is closely linked to metabolic disorders such as obesity, dyslipidemia, and hepatic steatosis. This study investigated the anti-thrombotic and metabolic effects of KoreaGinseng F Max (KGF), a standardized extract rich in ginsenosides, in high-fat diet (HFD)-induced obese rats, and complementary in silico analyses were used to explore putative molecular targets and pathways. The extract was standardized to contain 36.97 mg/g of ginsenosides Rg1, Rb1, and Rf. Male rats were administered KGF (50, 100, or 200 mg/kg) orally for six weeks. KGF significantly improved lipid profiles by reducing serum triglycerides, total cholesterol, and low-density lipoprotein (LDL) levels. Histological analysis revealed a dose-dependent reduction in hepatic steatosis and adipocyte size. Potential anti-thrombotic activity was evaluated using a FeCl_3_-induced carotid artery thrombosis model, with aspirin (30 mg/kg) included as a positive control. KGF200 delayed thrombus formation and produced a carotid blood flow pattern comparable to that observed in the aspirin-treated group, without significant alterations in serum ALT, AST, BUN, or creatinine levels. To further generate mechanistic hypotheses, complementary in silico analyses, including target prediction, GO/KEGG enrichment, network analysis, and molecular docking, were performed using the marker compounds. Eight overlapping genes, including STAT3, PTAFR, VEGFA, FGF2, HPSE, IL2, HSP90AA1, and LGALS3, associated with thrombotic regulation were identified. Pathway analysis suggested that PI3K–Akt signaling, calcium signaling, Th17 cell differentiation, and proteoglycan/ECM-related signaling may represent putative pathway-level mechanisms underlying the observed protective effects. Molecular docking suggested possible interactions between the marker ginsenosides and several predicted hub targets. Collectively, these findings suggest that KGF may have potential for further investigation as a natural product-derived material for improving HFD-associated metabolic and thrombotic dysfunction, while the predicted multi-target and multi-pathway effects require further experimental validation.

## 1. Introduction

Despite increased awareness and efforts to understand and confront its causes, the incidence of obesity continues to grow globally due to preferences for high-fat foods, especially those high in saturated fat. A high-fat diet (HFD) causes triglyceride accumulation in many tissues, particularly adipose tissue, contributing to metabolic disorders such as complex and chronic cardiovascular disease [1,2]. Notably, cardiovascular disease (CVD) attributed to hypertension, hyperlipidemia, and atherosclerosis are among the top five causes of global morbidity and mortality in both industrialized and developing countries. The World Health Organization (WHO) anticipates the annual CVD mortality to increase from 17.5 million in 2012 to 22.2 million in 2030 [3]. It is well-established that the incidence of CVD is closely associated with hyperlipidemia, a condition characterized by significant increases in total cholesterol (TC), triglycerides (TGs), and low-density lipoprotein (LDL), as well as a decrease in high-density lipoprotein (HDL) [4].

Hyperlipidemia is often accompanied by platelet hyperactivity, hypercoagulability with increased factor VII, and hypofibrinolysis with increased plasminogen activator inhibitor PAI-1. It also plays a crucial role in the pathogenesis of CVD, such as atherosclerosis [5,6]. The destruction of atherosclerotic plaques and the subsequent development of occlusive thrombus in the arteries is a critical process involved in the onset of atherothrombosis. The initial step of hemostatic and thrombotic processes is platelet activation. Therefore, the development of platelet-rich thrombi is a major cause of acute cardiovascular complications [7,8], leading to the development of numerous therapies aimed at suppressing platelet function [9].

Accumulating evidence suggests that oxidative stress is one of the important mechanisms involved in obesity-associated metabolic and cardiovascular disorders [10,11]. Excessive production of reactive oxygen species (ROS) induced by the consumption of a high-fat diet contributes to endothelial dysfunction, platelet hyperreactivity, vascular inflammation, and hepatic lipid accumulation, thereby promoting the progression of atherosclerosis and thrombotic complications [11,12]. Oxidative stress is also closely linked to chronic low-grade inflammation and dysregulated lipid metabolism observed in obesity-related cardiovascular disease [13]. Although oxidative stress has been implicated in the pathogenesis of obesity-associated cardiovascular disease, the present study primarily aimed to evaluate the anti-thrombotic and metabolic effects of KGF rather than directly assess oxidative stress-related biomarkers.

In addition to oxidative stress and platelet hyperreactivity, recent studies have highlighted the importance of macrophage dysfunction and impaired efferocytosis in the progression of chronic vascular inflammation and atherothrombotic disease. Defective clearance of apoptotic cells promotes plaque instability and increases thrombotic risk. Recent studies have further suggested that epigenetic regulators, including RBPJ, contribute to macrophage efferocytosis and vascular homeostasis [14].

Aspirin, one of the most widely used drugs in the world, is commonly used for preventing CVD. Low-dose aspirin regimens (≥30 mg/day) can effectively suppress platelet aggregation without affecting important endothelial cell functions [15]. The anti-thrombotic effects of aspirin are due to its pharmacological mechanism of irreversible inactivation of the cyclooxygenase (COX) enzyme and related suppression of prostaglandin production [16]. The main disadvantage of continuous aspirin use is the risk of major gastrointestinal and, in rare cases, intracranial bleeding, because aspirin is acidic and stimulates the stomach to increase gastric acid secretion. Notably, these bleedings can occur at any dose [17]. Other side effects, such as hypertension and renal impairment, can occur, usually in a dose-related manner. Moreover, a recent meta-analysis that studied the relationship between regular aspirin use and CVD prevention in adults aged 50 years and older (*n* = 11,392 unweighted) found that aspirin does not provide a clear benefit for primary CVD events in people over 75 years of age [18]. Therefore, this has led to a search for alternative natural, safe, and efficacious agents which have a similar degree of efficacy without adverse effects associated with conventional drug medication. Oriental medicines or medicinal plants are an attractive alternative source of pharmacologically active compounds and drugs [19].

Ginseng is a perennial herb of the family Araliaceae, with seven species distributed globally, of which Korean ginseng (*Panax ginseng* C.A. Meyer) has been widely used in the formulation of tonics in Eastern Asia, including Korea, for over 2000 years in the belief that it is a panacea and promotes longevity [20]. Depending on the processing method, fresh *Panax ginseng* can be processed into white ginseng (prepared by drying) or red ginseng (prepared by steaming followed by drying). It is well-established that various ginseng compounds, including ginsenosides, vitamins, polysaccharides, and essential oils, have clinical and pharmacological effects, including anti-cancer (anti-metastatic) properties [21], modulation of immune functions [22], promotion of hematopoiesis [23], and fatigue alleviation [24]. In Korea, the Ministry of Food and Drug Safety (MFDS) has officially recognized Korean ginseng as a functional ingredient with approved health claims, including immune enhancement, fatigue relief, maintenance of bone health, and liver function improvement. According to the Korean MFDS, the marker compounds designated for standardization of Korean ginseng are ginsenoside Rg1 and Rb1. In addition, there have been many reports on Korean ginseng for preventing platelet aggregation and thereby improving blood flow. Clinical studies have shown that Korean Red Ginseng treatment can be beneficial for individuals with CVD, which is thought to be due to its ability to inhibit platelet aggregation rather than anticoagulation [25,26]. Another study reported that phenolic acid extract from ginseng powerfully inhibited the palmitate-induced accumulation of lipids and increased endothelial cell viability by enhancing the phosphorylation of the phosphatidylinositol 3-kinase/protein kinase B/endothelial nitric oxide synthase (PI3K/Akt/eNOS) signaling pathway [27]. Moreover, metabolomics studies showed that Korean Red Ginseng intake lowers blood pressure in patients with prehypertension [28] and rats [29] by decreasing blood lysophosphatidylcholine and increasing dihydrobiopterin levels. These results suggest that ginseng intake has an important anti-thrombotic effect that may be beneficial for people with thrombotic problems and CVD.

In addition to its anti-thrombotic and metabolic activities, Korean ginseng has been reported to exert antioxidant effects through the modulation of oxidative stress-related pathways, including suppression of reactive oxygen species production and enhancement of endogenous antioxidant defense systems [30]. These antioxidant properties may contribute to the vascular and metabolic protective effects of ginseng under obesity-associated pathological conditions [27,30].

The present study was designed to investigate the anti-thrombotic effects and concentration-dependent efficacy of Korean ginseng extracts (KoreaGinseng F Max; KGF) to identify marker compounds for quality control, and to examine their potential mechanisms using complementary in silico analyses. The KGF used in the present study is a standardized extract prepared from white Korean ginseng. To avoid taxonomic confusion with *Panax quinquefolius*, we additionally propose ginsenoside Rf—an exclusive component of *Panax ginseng*—as a distinguishing marker [31]. Accordingly, ginsenosides Rg1, Rb1, and Rf were designated as the marker compounds for quality assessment in this study and were further used for in silico analysis.

## 2. Materials and Methods

### 2.1. Preparation of Korean Ginseng Extracts

White ginseng, produced from 4-year-old *Panax ginseng*, was obtained from Daedong Korea Ginseng Co. (Geumsan-gun, Republic of Korea). A voucher specimen (Lot 24012201) was deposited at the Herbarium of the Department of Herbal Crop Research, National Institute of Horticultural and Herbal Science, Rural Development Administration, Eumseong, Republic of Korea. For the extraction process, 200 kg of white ginseng was sequentially extracted using 60% (*v*/*v*) food-grade ethanol, followed by 40% (*v*/*v*) food-grade ethanol, and water. The sequential extraction procedure was established based on preliminary optimization experiments. This multi-step extraction process was designed to maximize the recovery of bioactive constituents with a broad range of polarities while maintaining the major ginsenoside content. The extract was concentrated under vacuum until it reached a soluble solids content of above 65 Brix to obtain the ginseng extracts (KoreaGinseng F Max, KGF). All extraction and concentration procedures were conducted in a facility certified for Good Manufacturing Practice (GMP). For subsequent experiments, KGF was lyophilized using a freeze dryer (EYELA, Tokyo, Japan) to make powder samples.

### 2.2. High-Performance Liquid Chromatography Analysis of Marker Compounds

Marker compounds in KGF were analyzed using HPLC. Powdered KGF was re-dissolved using 10% acetonitrile (*v/v*), diluted to 5000 ppm, and injected into the Agilent 1200 Series HPLC system (Santa Clara, CA, USA). Ginsenoside Rg1, Rb1, and Rf as marker compounds in the extract were quantified. The UV detection wavelength was set to 203 nm. Chromatographic separations were carried out on a YMC Pack ODS-AM column (4.6 × 250 mm, 5 μm; YMC Co., Ltd., Kyoto, Japan). The column oven temperature was maintained at 50 °C, and the mobile phases were composed of solvent A (water) and solvent B (acetonitrile). The elution conditions were as follows: 0–6.5 min, 27% B; 6.5–10 min, 28% B; 10–15 min, 30% B; 15–24 min, 30% B; 24–25 min, 44% B; 25–32 min, 44% B; 32–33 min, 72% B; 33–35 min, 72% B; 35–36.5 min, 90% B; 36.5–40 min, 90% B; 40–45 min, 27% B; 45 min, 27% B. The injection volume was 10 μL and the flow rate was changed from 0.8 to 0.6 mL/min at 10 min, and from 0.6 to 0.8 mL/min at 33 min.

### 2.3. Experimental Animals

All experiments were conducted in accordance with the National Institutes of Health Guide for the Care and Use of Laboratory Animals (NIH Publication No. 80-23, revised 1996). The experimental protocols were approved by the Institutional Animal Research Ethics Committee of Eulji University (Approval No. EUIACUC23-22). Six-week-old male Sprague-Dawley rats (weighing 180–220 g) were purchased from Samtako Inc. (Osan, Republic of Korea) and were randomly housed in cages under a standard 12 h light/dark cycle (lights on from 7:00 am) and constant temperature (22 ± 2 °C).

**Figure 1:**
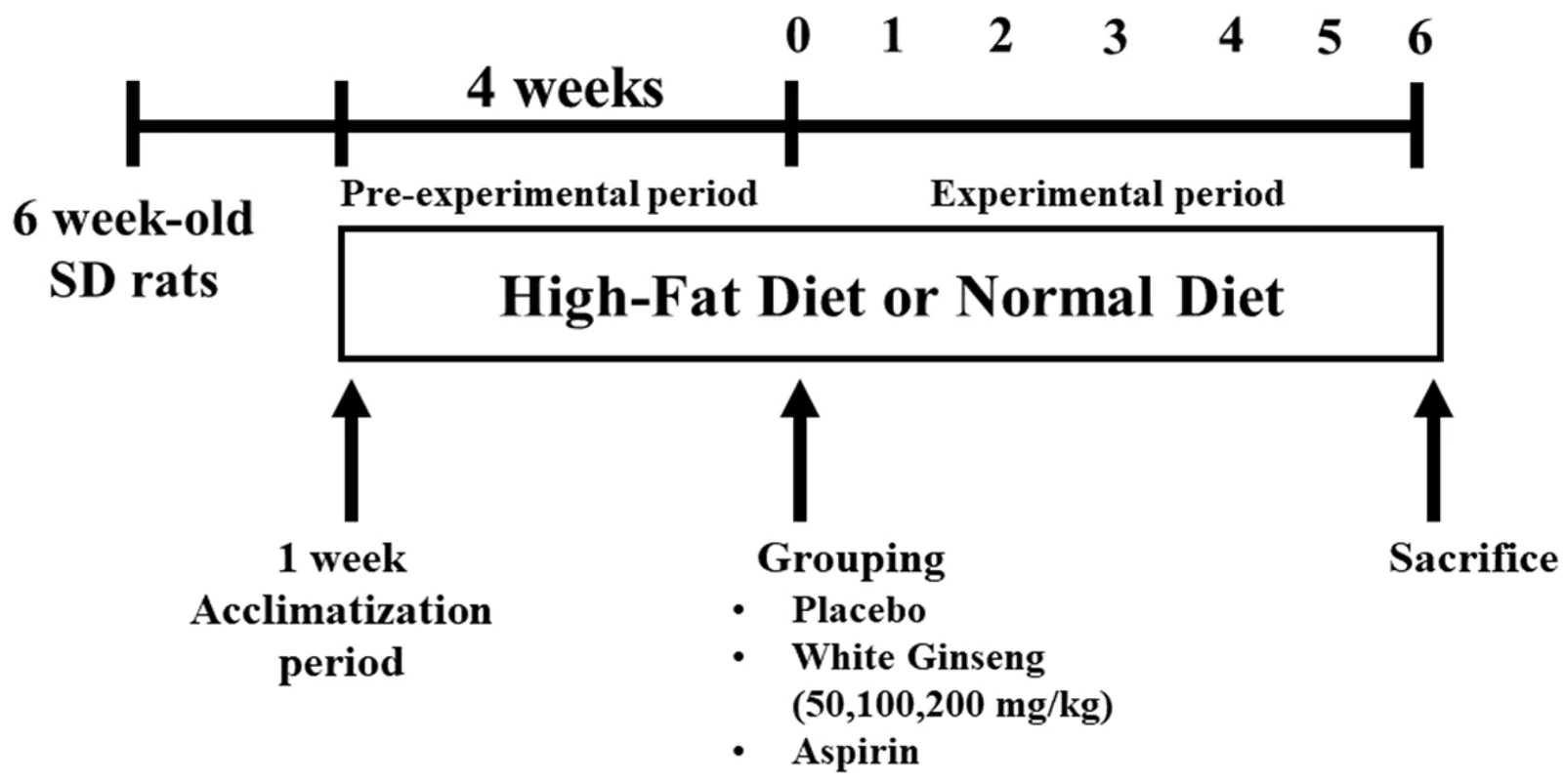
Schematic diagram of the experimental protocol. Rats were acclimated for 1 week and fed a high-fat diet (HFD, 60% fat diet) for 4 weeks (pre-experimental period) to induce an animal model of obesity. The animals on the HFD for 4 weeks were weighed and randomly assigned to five groups (placebo (HFD), KGF 50 mg/kg (KGF 50), KGF 100 mg/kg (KGF 100), KGF 200 mg/kg (KGF 200), and aspirin (ASP)) so that the mean body weight was approximately equal between groups. The control group (CONT) was fed a standard chow diet until the end of the experiment. The animals were sacrificed after 6 weeks.

Figure 1 shows the experimental design and timeline of this study. An HFD-induced obesity model was established to evaluate the metabolic effects of KGF and its potential anti-thrombotic activity under obesity-associated metabolic conditions. Rats were housed on a standard chow diet (#S-01010, Samtako Bio Korea, Osan, Republic of Korea) and had access to water ad libitum for a 1-week acclimatization period. The composition and caloric content of the control and high-fat diets are summarized in Supplementary Table S3. After weighing each individual at the end of the acclimatization period, the rats were divided into control (*n* = 9) and obese (*n* = 45) groups so that the mean weight of the two groups was approximately equal. The control (CONT) group received a standard chow diet throughout the pre-experimental (4 weeks) and subsequent experimental (6 weeks) periods. The obese group was fed a 60% fat diet (high-fat diet [HFD], #D12492; Research Diets Inc., New Brunswick, NJ, USA) to induce obesity. Four weeks after starting the HFD feeding (pre-experimental period), each individual was weighed and the obese group was further subdivided into five subgroups—placebo, KGF 50, KGF 100, KGF 200, and aspirin—so that the mean weight per group was similar. Rats in the KGF 50, KGF 100, and KGF 200 groups were given 50 mg/kg, 100 mg/kg, and 200 mg/kg of KGF dissolved in 1 mL of distilled water thrice a week orally by gavage for 6 weeks. The placebo group (HFD group) and aspirin group (ASP group) were treated in the same manner as above, with 1 mL of distilled water and 1 mL of aspirin (30 mg/kg, #PHR1003-1G, Sigma-Aldrich Co., St. Louis, MO, USA)—a dose selected based on previous studies [32,33]—dissolved in distilled water, respectively, for 6 weeks. Each cage contained three rats. Rat food consumption and body weight were measured weekly.

A total of nine rats per group were used in this study (total number = 54), of which seven rats per group were used for biochemical analysis, ELISA, and histological analysis, and two rats per group were used for blood flow measurement using laser Doppler flowmetry.

### 2.4. Measurement of Average Daily Food Consumption

Food intake was quantified once a week during the 6-week experimental period. A total of 150 g of HFD or a standard chow diet per individual cage was provided on Wednesdays at 14:00, and any remaining amount was measured after 24 h (Thursdays at 14:00) using an automatic electronic balance (Sartorius, Göttingen, Germany). The amount of food consumed per cage was divided by the number of animals (*n* = 3) in the cage, and this was considered to be the individual mean daily food consumption (g/day/rat). Caloric intake was calculated by multiplying the food consumption by the caloric values of the diets used.

### 2.5. Sample Collection and Relative Organ Mass Determination

At the end point of the experiment, 12 h fasted rats were anesthetized via intraperitoneal injection of a mixture of ketamine (70 mg/kg; Yuhan Co., Seoul, Republic of Korea) and xylazine (Rompun, 8 mg/kg; Bayer Korea Co., Seoul, Republic of Korea). After the abdominal cavity was opened, blood samples were collected through the abdominal aorta and centrifuged at 3000 rpm for 15 min at 4 °C. Serum was separated and stored at −80 °C until analysis. The liver and adipose tissue of the left epididymis were carefully removed, cleaned briefly in cold saline, and weighed. The relative organ weight, i.e., the ratio of the weight of an organ to the weight of the whole body, was calculated as follows: (absolute organ weight/body weight at sacrifice) × 100. For histological analysis, the largest lobule of the liver and the epididymal fat pads were sectioned and fixed in 4% paraformaldehyde.

### 2.6. Biochemical Analysis

Serum levels of triglycerides (TGs), total cholesterol (TC), high-density lipoprotein (HDL), and low-density lipoprotein (LDL) were measured by standard colorimetric methods. The concentrations of serum alanine aminotransferase (ALT), aspartate aminotransferase (AST), blood urea nitrogen (BUN), and creatinine were quantified by kinetic methods. The available diagnostic kits were purchased from Asanpharm Co., Ltd. (Seoul, Republic of Korea).

All the biochemical analyses were performed using a semi-automated biochemical analyzer (Humalyzer 2000, Human GmbH Diagnostica, Wiesbaden, Germany) according to the manufacturer’s instructions. The inter-laboratory precision and accuracy of the biochemical analysis was confirmed by regular calibration using reference standards provided in the kits.

### 2.7. Analysis of Plasma Leptin, Adiponectin, and ACE Levels

The plasma leptin, adiponectin, and angiotensin-converting enzyme (ACE/CD143) levels were determined using a commercial enzyme-linked immunosorbent assay (ELISA) kit (R&D system Inc., Minneapolis, MN, USA) according to the manufacturer’s instructions. The Rat Leptin ELISA Kit (Catalog #: NIB00B), Rat Total Adiponectin ELISA Kit (Catalog #: RRP300), and Mouse ACE-2 DouSet ELISA Kit (Catalog #: DY3437-05) were used for the leptin assay, adiponectin assay, and ACE assays, respectively. All these experiments were performed by the ELISA reader system (MRX A2000, KLAB Co., Ltd., Seoul, Republic of Korea).

### 2.8. Histological Analysis

The following routine methods were used for histology and morphometry. Liver and epididymal adipose tissues, which had been fixed in 4% paraformaldehyde, were embedded in paraffin. Paraffin sections were cut at a thickness of 4 µm and stained with hematoxylin and eosin (H&E). The sections were dehydrated through a graded ethanol series, cleared in xylene, and coverslipped with Permount (Fisher Scientific, Pittsburgh, PA, USA). Light micrographic images were observed and photographed using a LEICA DM6M microscope (Leica Microscope System, Wetzlar, Germany) equipped with a LEICA DFC295 digital camera (Leica Microscope System).

The adipocyte cell size was measured in ten randomly selected microscopic areas (20× objective) from five randomly sampled specimens (100 µm intervals across the sample tissue) from individual animals. The mean diameter and area of adipocytes were estimated by dividing the sum of the diameter and area of each adipocyte measured in each selected microscopic area by the total number of selected adipocytes (*n* = 500). Quantification of liver steatosis was performed in three randomly selected microscopic areas (20× objective) per specimen from ten specimens (500 intervals across the sample tissue) systematically collected at random from individual animals. Qupath (version 0.3.2, University of Edinburgh, Edinburgh, UK) open-source software was used to score the degree of steatosis. The software allows accurate discrimination of empty vesicles (10 µm^2^ in size or less) contained in the selected microscopic area. The steatosis score was expressed as a percentage of the number of steatotic hepatocytes measured divided by the total number of hepatocytes present in the entire observation area. Two observers, blind to the experimental design, independently performed measurements on the same microscopic fields and the mean value was used for analysis.

### 2.9. Measurement of Blood Flow

After deep anesthesia with an intraperitoneal injection of a mixture of ketamine and xylazine, the rats were placed on a heating pad in the supine position. A midline incision was made in the neck, and the right common carotid artery (CCA) was carefully dissected from the vagus nerve and surrounding fascia under an operating microscope. CCA blood flow was monitored and recorded for 10 min using a real-time microcirculation imaging system (PeriCam PSI HR, Perimed, Stockholm, Sweden). Arterial thrombosis was then induced by wrapping the CCA with Advantec filter paper No.2 (Toyo Roshi Kaisha Ltd., Tokyo, Japan) (3 × 5 mm) soaked in 30% FeCl_3_ solution for 20 min. Changes in blood flow caused by thrombus formation in the CCA were measured at 5 min intervals, and each measurement was performed for 1 min 30 s.

### 2.10. Collection of Ginsenosides and Anti-Thrombotic Targets

All potential targets of ginsenosides Rb1, Rg1, and Rf were predicted using SwissTargetPrediction (https://www.swisstargetprediction.ch/, accessed on 6 April 2026, 2019 version) [34], limited to Homo sapiens. Anti-thrombotic-related genes were obtained from the GeneCards database (https://www.genecards.org/, accessed on 8 April 2026) to identify overlapping targets between compound-derived and disease-associated gene sets. Homo sapiens was used because SwissTargetPrediction and GeneCards are primarily human-gene-based databases; therefore, these in silico results should be interpreted as exploratory predictions rather than direct rat-specific mechanistic evidence.

### 2.11. Gene Ontology and Kyoto Encyclopedia of Genes and Genomes Enrichment Analyses

Overlapping genes between ginsenosides and anti-thrombotic-related genes were identified using Venny (https://bioinfogp.cnb.csic.es/tools/venny/, accessed on 8 April 2026, v2.1.0) and analyzed for Gene Ontology (GO) and Kyoto Encyclopedia of Genes and Genomes (KEGG) enrichment using Metascape (v3.5) [35], limited to Homo sapiens with *p* < 0.05. Enrichment results were visualized using SRplot (https://www.bioinformatics.com.cn/, accessed on 8 April 2026). The relationships among enriched GO terms, key genes, and KEGG pathways were further integrated into a functional network and visualized using Cytoscape (v3.10.3). In this network, nodes were categorized into GO terms, key genes, and KEGG pathways, and the selected key genes and representative predicted pathways were highlighted in red. In addition, the shared genes were mapped onto four representative KEGG pathways, namely the PI3K–Akt signaling pathway, calcium signaling pathway, Th17 cell differentiation, and proteoglycan/ECM-related signaling, using SRplot [36].

### 2.12. Molecular Docking and Construction of a Proposed Mechanistic Hypothesis

Hub target proteins were identified using the AD3.io library and platform, including STAT3 (P40763; signal transducer and activator of transcription 3), PTAFR (P25105; platelet-activating factor receptor), VEGFA (P15692; vascular endothelial growth factor A), FGF2 (P09038; fibroblast growth factor 2), HPSE (Q9Y251; heparanase), IL2 (P60568; interleukin-2), HSP90AA1 (P07900; heat shock protein 90 alpha family class A member 1), and LGALS3 (P17931; galectin-3). The three marker ginsenosides (Rb1, Rg1, and Rf) were retrieved from PubChem, and ligand structures were converted into PDB format using Open Babel. Molecular docking simulations were performed using AD3.io (www.ad3.io, accessed on 22 April 2026; Copyright 2026 Arontier Co., Ltd.), and the binding affinities between the ligands and target proteins were calculated using AK-Score2 [37]. In the AD3.io platform, lower or more negative docking scores were interpreted as more favorable predicted binding. Some compound–protein pairs produced abnormally large positive scores, which were regarded as unsuccessful or energetically unfavorable docking results. For heatmap visualization, these positive values were converted to 0 kcal/mol and were not interpreted as evidence of stable binding. A proposed mechanistic hypothesis illustrating the relationship between the observed in vivo findings and complementary in silico predictions was constructed using BioRender.

### 2.13. Statistical Analysis

All data were presented as mean ± standard deviation (SD). Statistical differences between groups were analyzed using the Kruskal–Wallis H test or one-way analysis of variance followed by Dunnett’s multiple comparison test (GraphPad Prism 8.0; GraphPad Software, Inc., La Jolla, CA, USA). A *p*-value of <0.05 was considered statistically significant.

## 3. Results

### 3.1. HPLC Analysis of Marker Compounds in KGF

**Figure 2:**
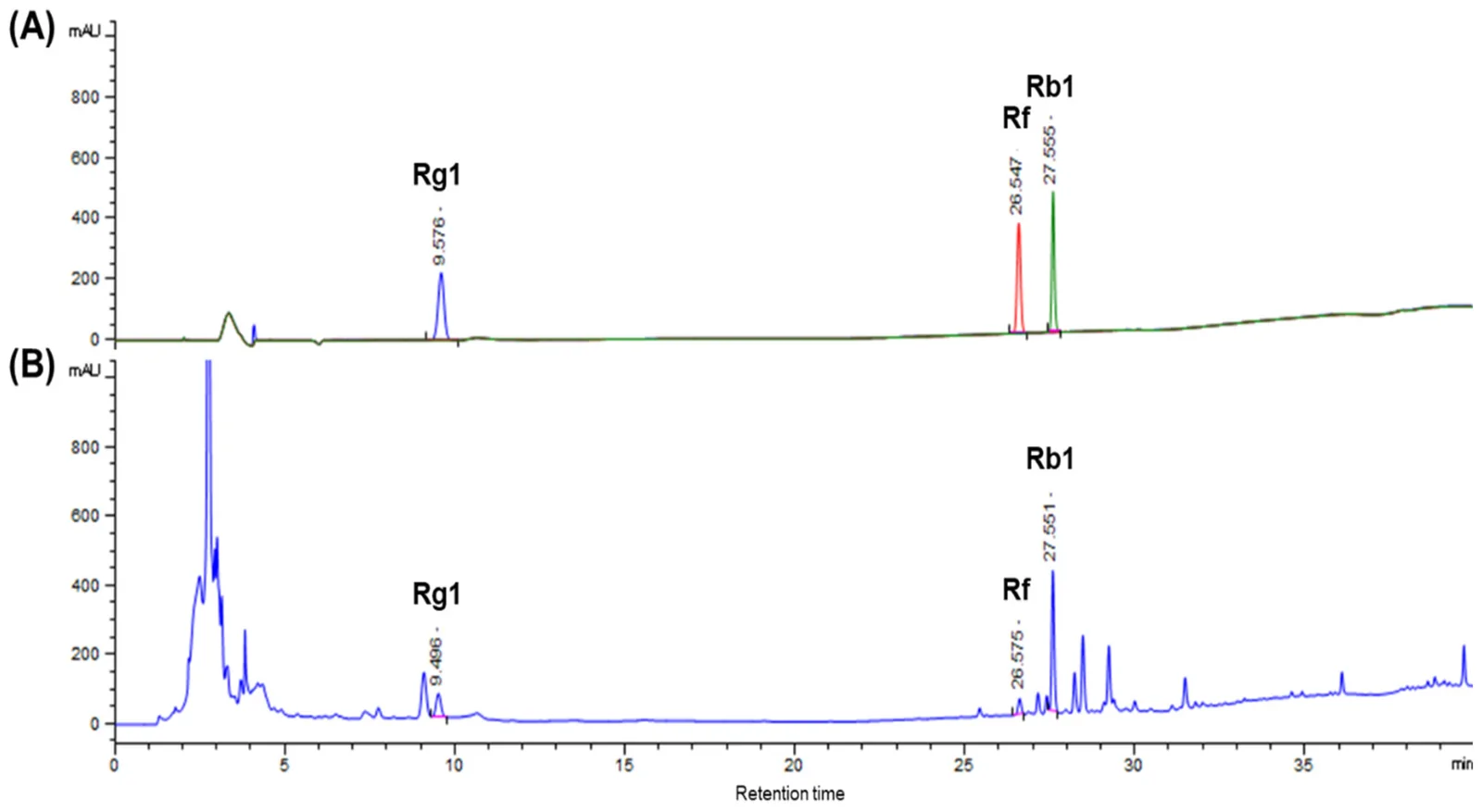
HPLC-UV chromatograms (203 nm) of standard overlay (**A**) and KGF (**B**). HPLC-UV, high-performance liquid chromatography with ultraviolet detection; KGF, KoreaGinseng F Max.

Ginsenoside Rg1, Rf, and Rb1 were analyzed as marker compounds using an HPLC system. Ginsenosides Rg1 and Rb1 have been established as standard marker compounds for white ginseng [38]. In addition, ginsenoside Rf, which serves to distinguish *Panax ginseng* from *P. quinquefolius* [31], was adopted in this study as an additional marker component. Three ginsenosides were quantified in the white ginseng extract. An overlay chromatogram of standard compounds and a chromatogram of white ginseng extract are shown in Figure 2. The three ginsenosides present in the extract were quantified. Total compounds totaled 36.97 mg/g in the extract. The mean concentrations of ginsenoside Rg1, Rf, and Rb1 were 7.03, 3.35, and 26.58 mg/g, respectively (Table 1).

### 3.2. Changes in Food Consumption and Weight Gain in HFD Rats Following KGF Administration

**Figure 3:**
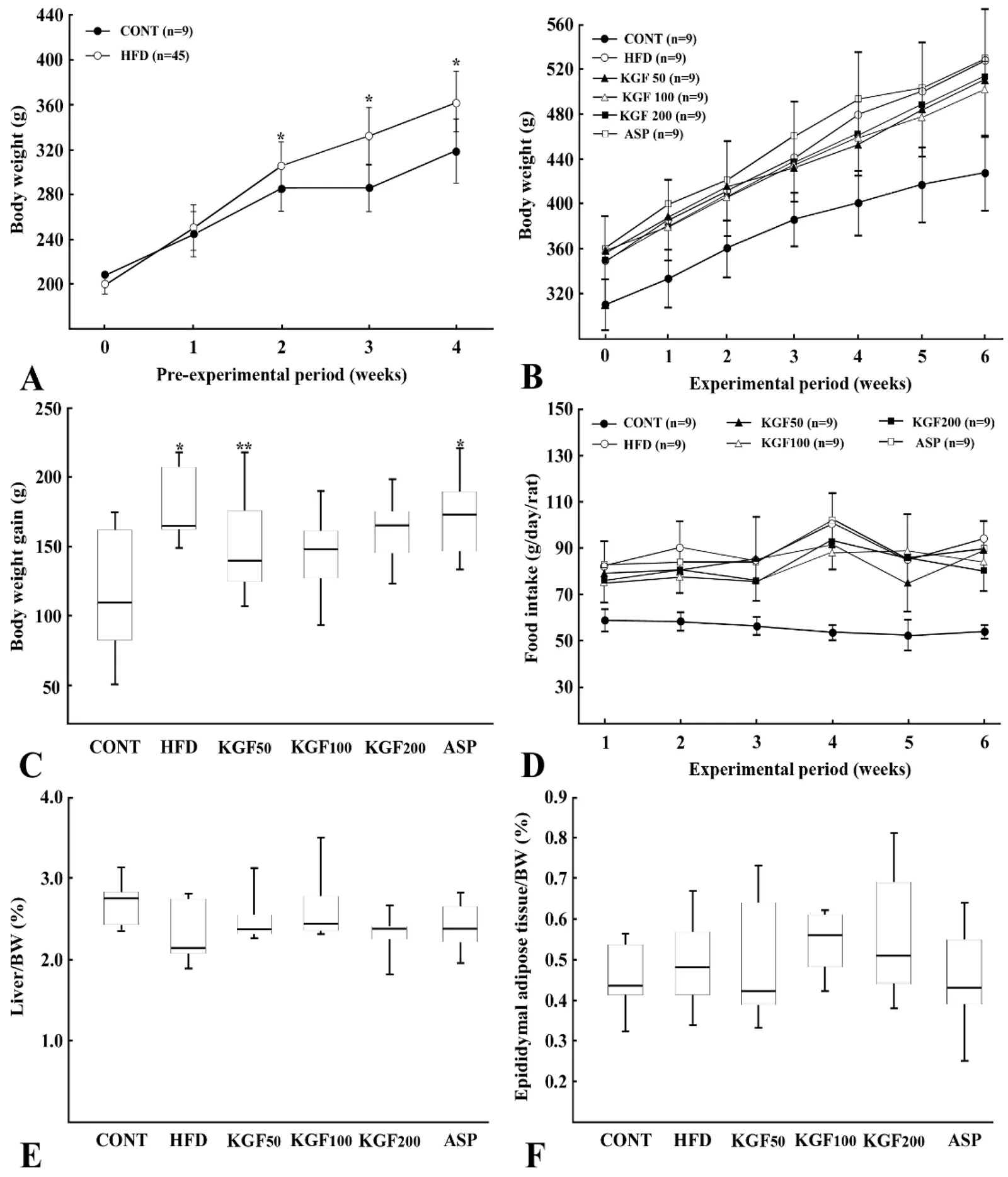
Effect of KGF administration on body weight, food consumption, and weight of the liver and epididymal adipose tissue. (**A**) Changes in mean Body weight of the experimental animals fed a standard chow diet (*n* = 9) and an HFD (*n* = 45) during the Pre-experimental period. (**B**) The average Changes in Body weight of the six experimental groups during the Experimental period. (**C**) Net weight changes and (**D**) daily Food consumption changes over the 6-week Experimental period. (**E**) Average weight of Liver and (**F**) epididymal adipose tissue of experimental animals at the end point of the experiment. All values were expressed as mean ± S.D. *p*-values were determined by one-way ANOVA. * *p* < 0.05 and ** *p* < 0.01; significant as compared with CONT group.

Rats fed the HFD showed a significant increase in body weight (from 2 weeks onwards) compared to rats fed the normal diet (CONT group) during the four-week pre-experimental period (Figure 3A and Table S1). A total of 45 obesity-induced rats were divided into five subgroups: those fed a continuous HFD without any drugs for the next 6 weeks of the experimental period (HFD group), those fed different concentrations of white ginseng extract with a HFD (KGF50 [50 mg/kg], KGF100 [100 mg/kg], and KGF200 [200 mg/kg] groups), and those fed aspirin (30 mg/kg) with a HFD (ASP group) (Figure 2).

During the subsequent 6 weeks of the experimental period, weight gain was significantly greater in the HFD group compared to the CONT group, while the KGF groups and ASP group had similar weight gain patterns to the HFD group (Figure 3B and Table S1). However, when analyzing the net weight change (weight at the end of the experiment–weight at the beginning of the experiment), the KGF groups tended to gain less weight than the HFD group. The HFD, KGF50, and ASP groups showed statistically significant differences compared to the CONT group, whereas the KGF100 and KGF200 groups did not show statistically significant differences compared to the CONT group (Figure 3C). We examined daily food consumption at weekly intervals. No substantial changes in food consumption were observed in any of the experimental groups throughout the study (Figure 3D). These findings suggest that neither KGF nor aspirin gavage altered caloric intake in the animals.

At the end of the experiment, the weights of the visceral organs, including the liver and epididymal adipose tissue, were measured. There were no significant differences in liver weight or epididymal fat weight among groups (Figure 3E,F).

### 3.3. KGF Administration Improves the Serum Lipid Levels

**Figure 4:**
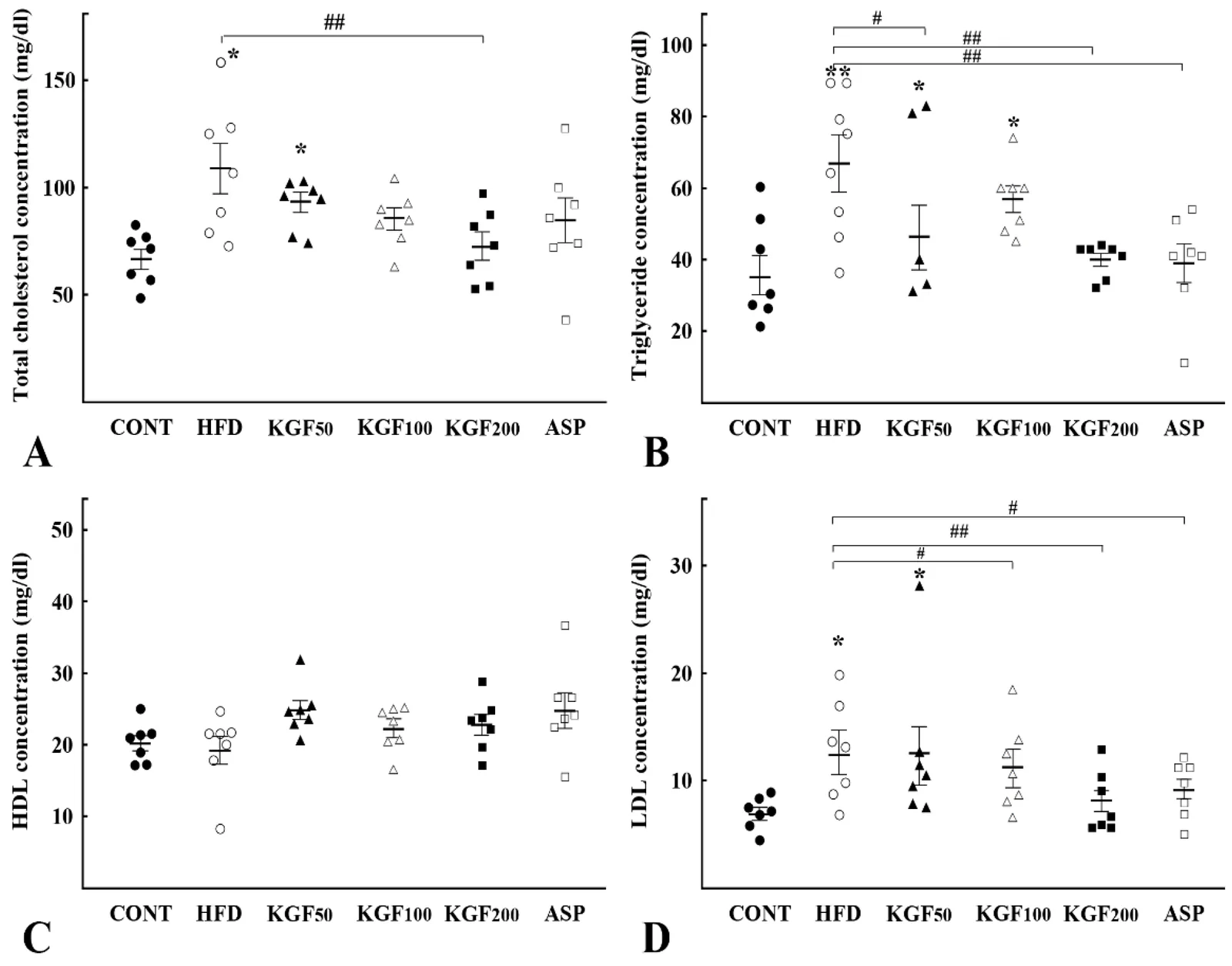
Effect of KGF administration on changes in serum lipid levels in the HFD-induced obese animal model. (**A**) Total cholesterol, (**B**) Triglyceride, (**C**) high-density lipoprotein (HDL), and (**D**) low-density lipoprotein (LDL) levels in whole blood were measured in the six experimental animal groups. All values were expressed as mean ± S.D. of seven rats. *p*-values were determined by one-way ANOVA. * *p* < 0.05 and ** *p* < 0.01, significant as compared with CONT group; ^#^ *p* < 0.05 and ^##^ *p* < 0.01, significant as compared with HFD group.

The serum lipid levels (TC, TGs, LDL, and HDL) of the experimental animals at the end point of the experiment were analyzed (Figure 4 and Table S2). In the case of TC, the HFD group showed higher levels compared to the CONT group, while the KGF200 group exhibited a statistically significant reduction compared to the HFD group. TG levels were significantly reduced in a dose-dependent manner relative to the HFD group (Figure 4A,B). In contrast, HDL levels showed no significant changes among the groups. However, LDL levels were significantly decreased in a dose-dependent manner in the KGF100 and KGF200 groups compared to the HFD group, and a significant reduction was also observed in the ASP group (Figure 4C,D).

### 3.4. Effect of KGF Administration on Lipid Metabolites

**Figure 5:**
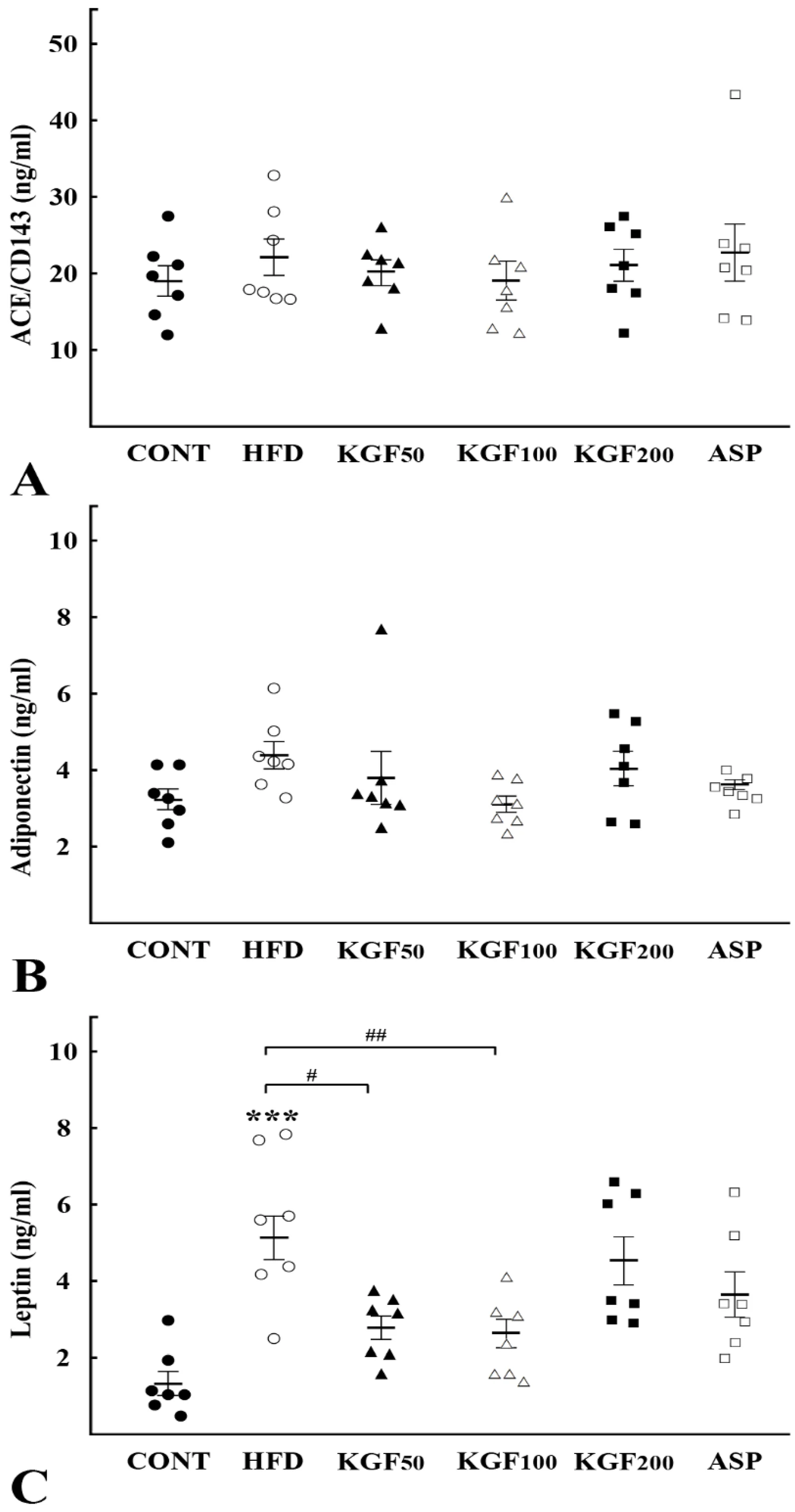
Effect of KGF administration on changes in serum (**A**) angiotensin-converting enzyme (ACE), (**B**) adiponectin, and (**C**) leptin levels in the HFD-induced obese animal model. All values were expressed as mean ± S.D. of seven rats. *p*-values were determined by one-way ANOVA. *** *p* < 0.001, significant as compared with CONT group; ^#^ *p* < 0.05 and ^##^ *p* < 0.01, significant as compared with HFD group.

The effect of KGF on serum levels of lipid metabolites, including ACE, adiponectin, and leptin, was investigated in each group at the end point of the experiment. As shown in Figure 5 and Table S2, the serum leptin levels were significantly elevated by about 5-fold in the HFD group compared to the CONT group, and oral KGF administration (KGF50 and KGF100 groups) significantly reduced the HFD-induced increase in serum leptin levels. However, KGF200 and ASP groups did not show significant differences in serum leptin levels compared to the HFD group. Meanwhile, serum ACE and adiponectin levels did not show statistically significant differences in any of the groups.

### 3.5. KGF Did Not Induce Hepatic and Renal Toxicity

**Figure 6:**
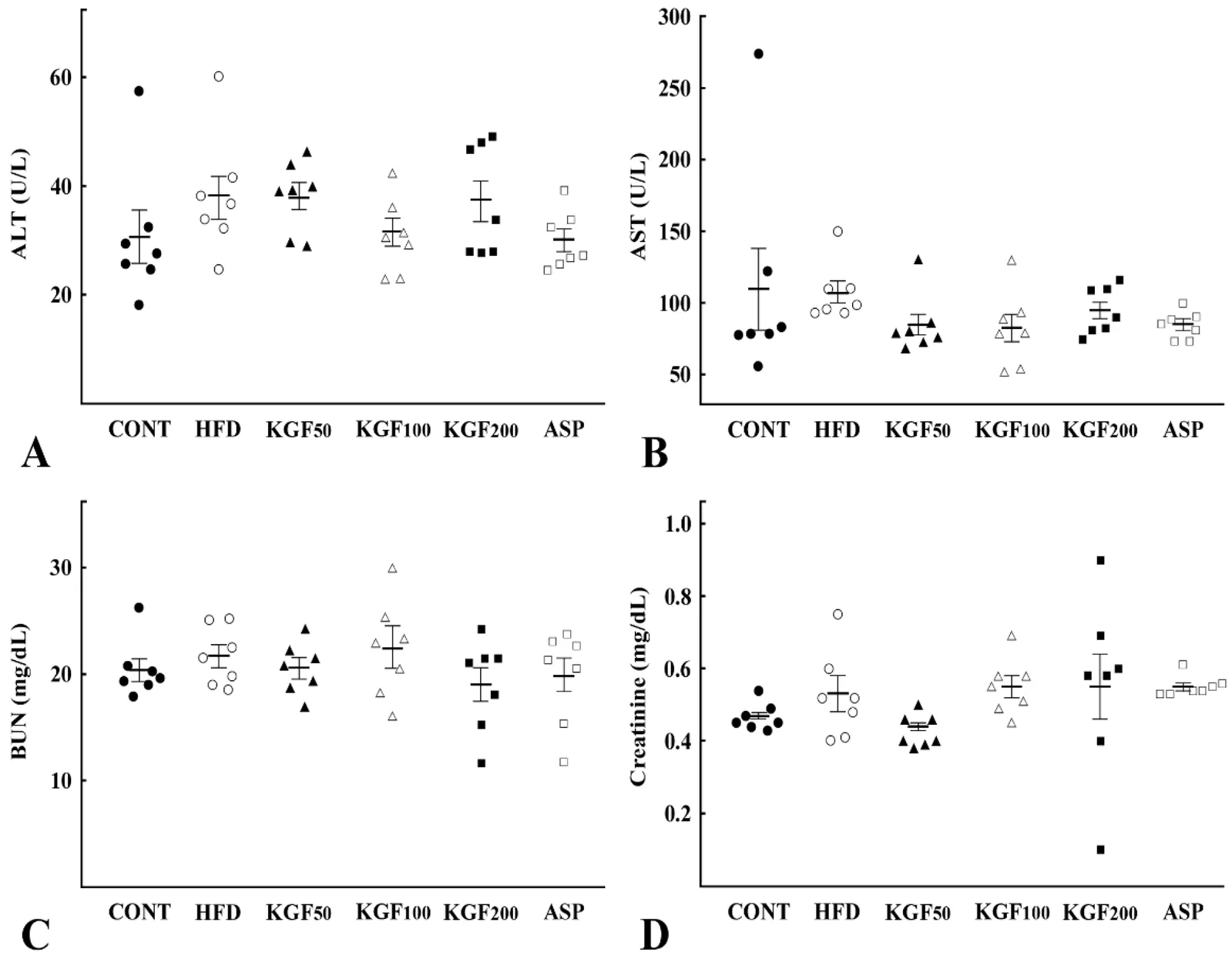
Effect of KGF administration on changes in serum (**A**) alanine aminotransferase (ALT), (**B**) aspartate aminotransferase (AST), (**C**) blood urea nitrogen (BUN), and (**D**) creatinine levels in the HFD-induced obese animal model. There were no statistical differences between the six groups for any of the measurements. All values were expressed as mean ± S.D. of seven rats. *p*-values were determined by one-way ANOVA.

To evaluate the effects of a HFD and/or KGF administration on serum biochemical markers of liver and kidney function, serum ALT, AST, BUN, and creatinine levels were measured and compared among the groups (Figure 6 and Figure S2). No significant differences were observed in serum ALT, AST, BUN, or creatinine levels among the six groups. These findings indicate that neither HFD nor KGF treatment significantly affected the serum biochemical markers of liver and kidney function under the present experimental conditions.

### 3.6. KGF Administration Reduces the Adipocyte Size and Liver Steatosis

**Figure 7:**
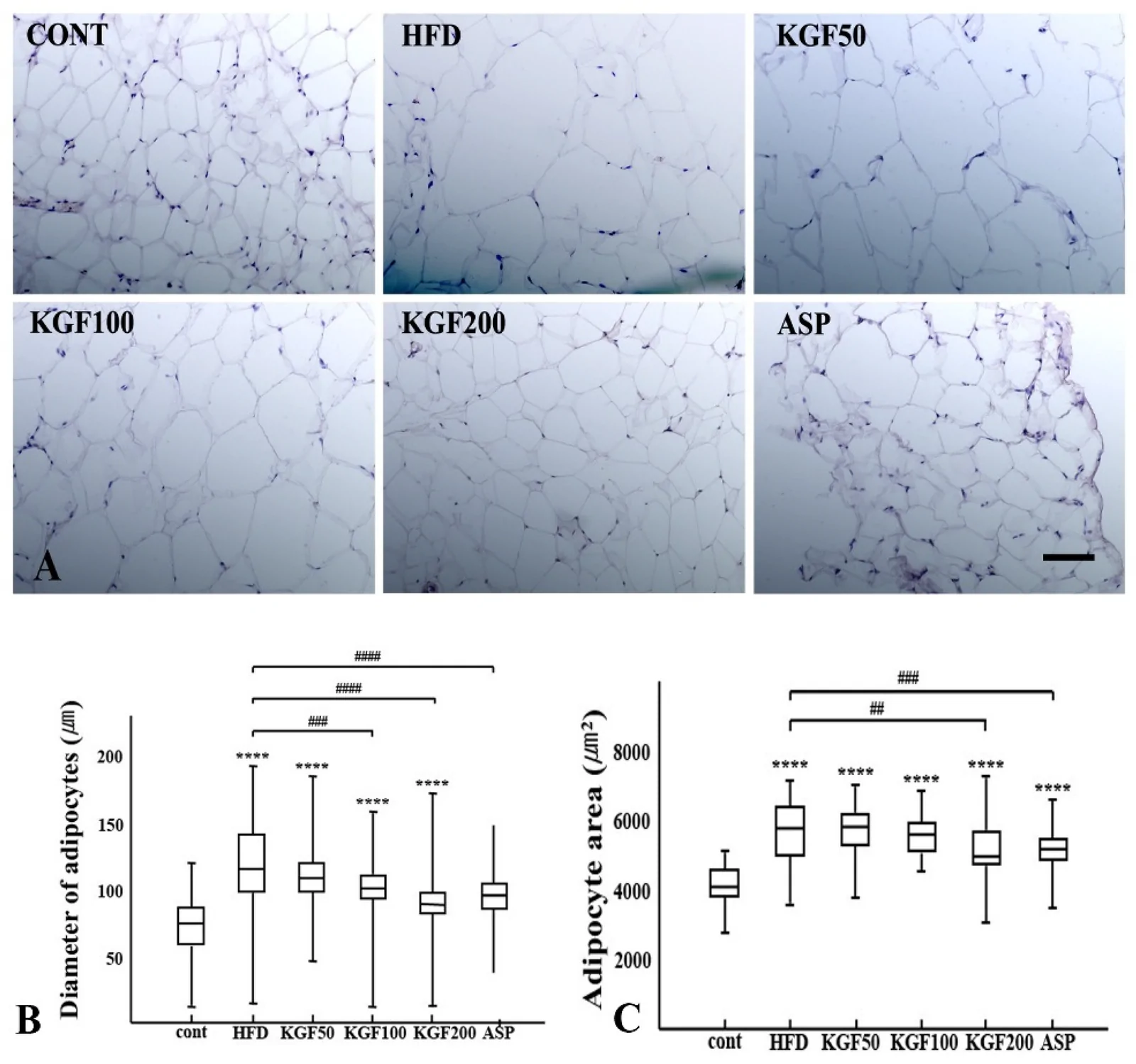
Effect of KGF administration on changes in adipocyte size. (**A**) Representative H&E-stained images of periepididymal adipocytes. (**B**) Mean diameter and (**C**) mean area of adipocytes in six experimental groups. Scale bars in (**A**) equal 100 μm. All values are expressed as mean ± S.D. of seven rats. *p*-values were determined by one-way ANOVA. **** *p* < 0.0001, significant as compared with CONT group; ^##^ *p* < 0.01, ^###^ *p* < 0.001, and ^####^ *p* < 0.0001, significant as compared with HFD group.

**Figure 8:**
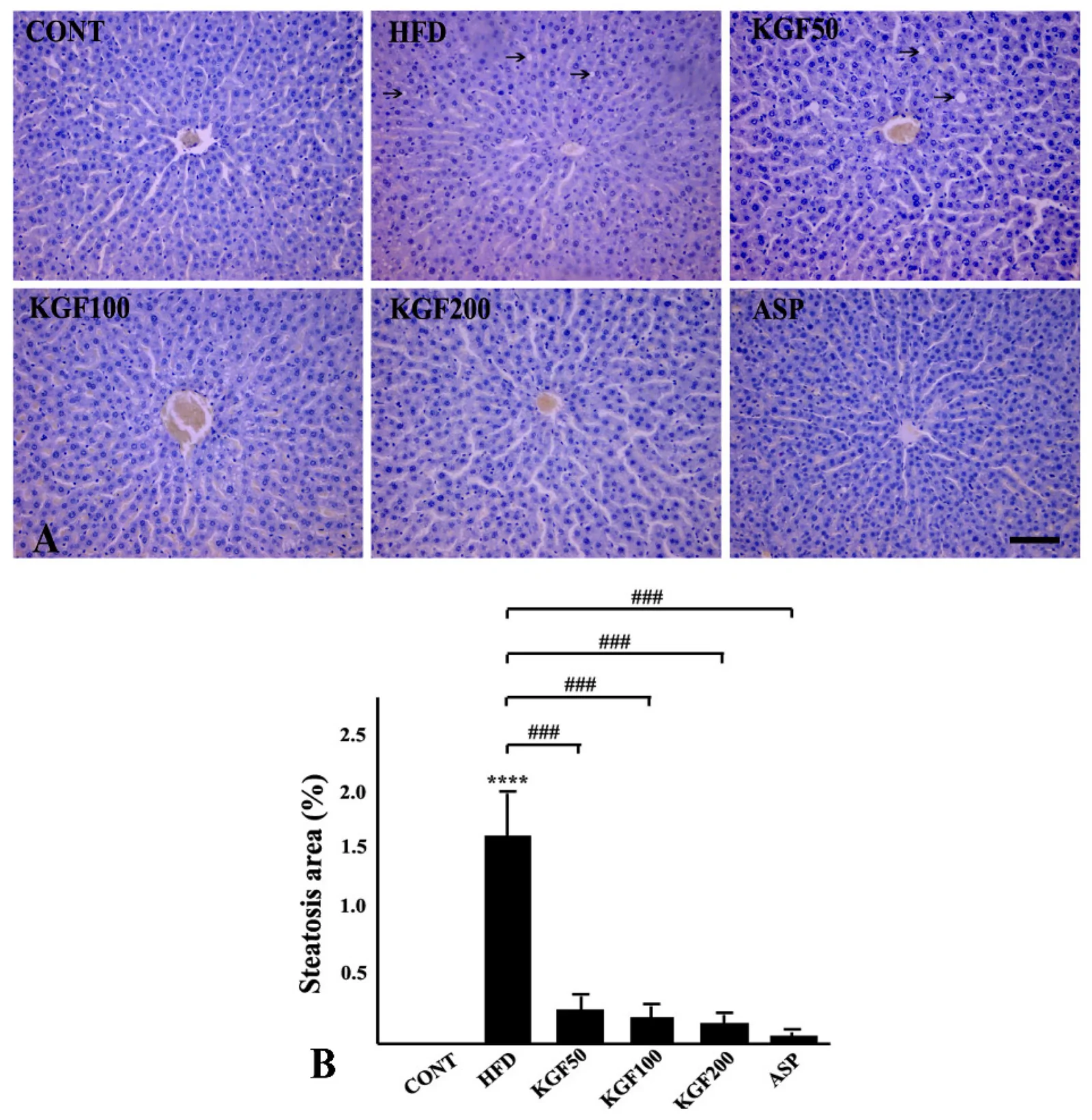
Effect of KGF administration on changes in hepatic steatosis. (**A**) Representative histological images of hepatocytes and (**B**) histomorphometric analysis of the steatosis area. The arrows in (**A**) indicate the area of steatosis. Scale bars in (**A**) equal 100 μm. All values are expressed as mean ± S.D. of seven rats. *p*-values were determined by one-way ANOVA. **** *p* < 0.0001, significant as compared with CONT group; ^###^ *p* < 0.001, significant as compared with HFD group.

The effects of KGF on changes in adipocyte size and on liver steatosis were investigated using histomorphometric studies (Figure 7 and Figure 8). The weight gains in epididymal adipose tissue and liver did not change between the six groups studied, as previously shown in Figure 3, but the mean diameter and area of adipocytes increased considerably in the HFD group compared to the CONT group and decreased markedly in the KGF groups in a dose-dependent manner and in the ASP group (Figure 7A–C).

Histological analysis of hepatic steatosis was also performed. In the HFD group, noticeable hepatic steatosis was induced by the deposition of lipid droplets and hypertrophic changes in the hepatocytes (Figure 8A). The steatosis score showed an approximately 1.5% change compared to the CONT group, while the KGF and ASP groups recovered to almost the same level as the CONT group (Figure 8B).

### 3.7. KGF Improves Blood Flow as Much as Aspirin

**Figure 9:**
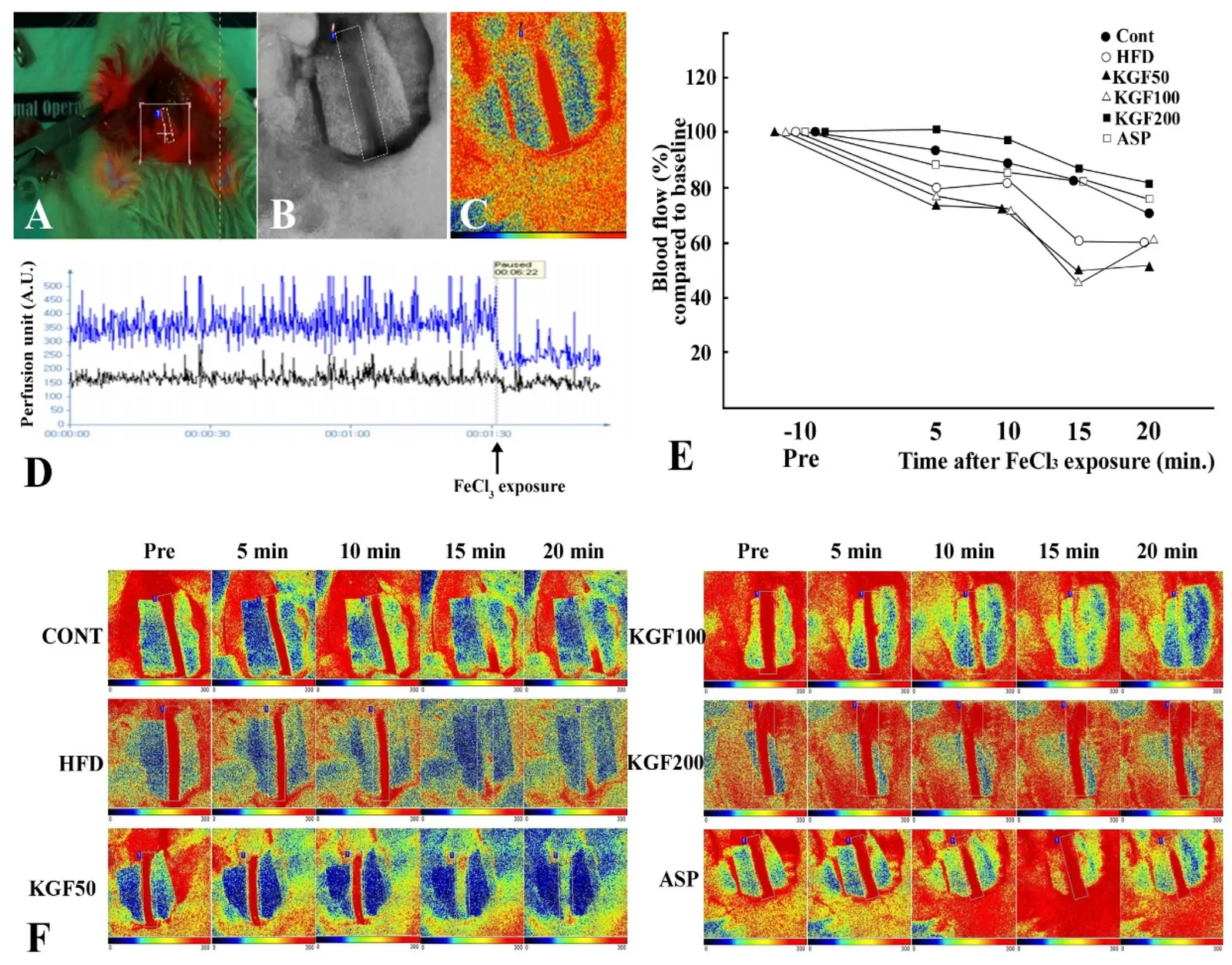
Time−course changes in arterial blood flow after FeCl_3_ application wrapped around the carotid artery using a real-time microcirculation imaging system. (**A**) Dissection image of a rat with a midline incision in the neck and an open common carotid artery (CCA). (**B**) Microscopic image of the CCA (dotted square) where the blood flow rate is measured, and (**C**) its Doppler blood flow image. (**D**) Graph showing the arterial perfusion unit before and after exposure to FeCl_3_. Note the significant decline in perfusion unit with FeCl_3_ exposure (arrow). (**E**) Graph of blood flow rates by group before and after FeCl_3_ exposure converted to percentage values. Mean blood flow units in the 10 min prior to FeCl_3_ exposure were standardized to 100%. (**F**) Serial Doppler blood flow images from each experimental group taken at 5 min intervals (5, 10, 15, and 20 min) after exposure to FeCl_3_. The carotid artery is red during the states at high blood flow, green at moderate blood flow, and blue at low blood flow.

Application of 30% FeCl_3_ around the common carotid artery for 20 min caused a progressive decrease in blood flow rate over time in all groups (Figure 9). In the HFD group, the blood flow rate was reduced by about 40% at 20 min after FeCl_3_ treatment, while in the KGF group it was reduced by about 20%. These findings suggest that oral administration of KGF200 for 6 weeks was associated with improved blood flow compared with the HFD group, showing a response comparable to that of aspirin. However, administration of 50 mg/kg of ginseng or 100 mg/kg of ginseng had no significant inhibitory effect on thrombus formation compared to rats fed the HFD alone.

### 3.8. Identification of Shared Genes

**Figure 10:**
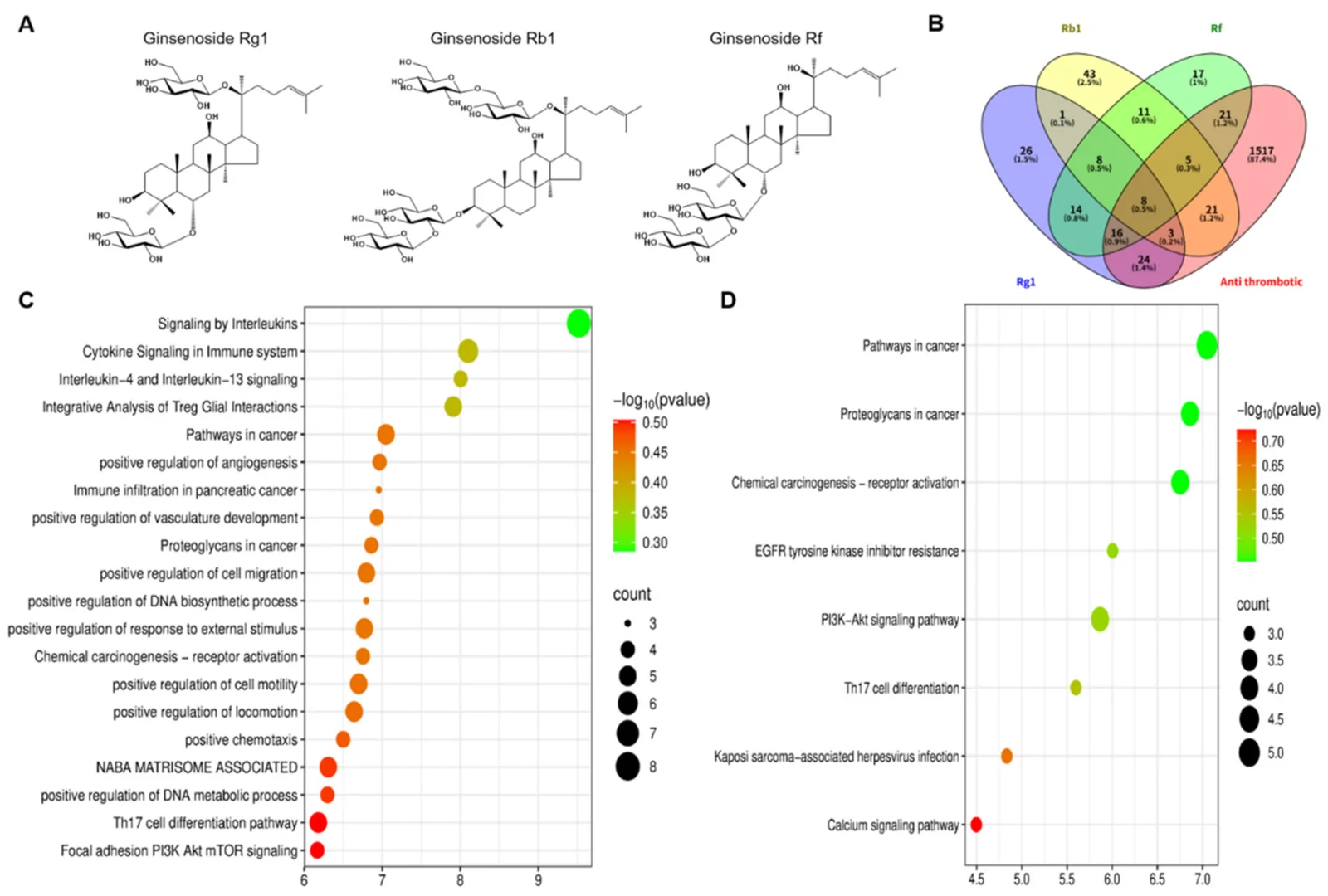
Network pharmacology analysis of white-ginseng-derived ginsenosides in anti-thrombotic-related egenes. (**A**) Chemical structures of the three major ginsenosides: Rg1, Rb1, and Rf. (**B**) Venn diagram showing the overlap between predicted targets of the three ginsenosides and anti-thrombotic-related genes. (**C**) GO biological process enrichment analysis of overlapping target genes, with the top 20 enriched terms visualized as a bubble plot. (**D**) KEGG pathway enrichment analysis of shared target genes associated with anti-thrombotic-related genes.

Potential genes associated with the ginsenosides Rg1, Rb1, and Rf were predicted using SwissTargetPrediction, resulting in 102, 102, and 102 candidate genes, respectively. The predicted targets of Rg1 were mainly distributed among secreted proteins, transcription factors, and transferases, whereas Rb1-associated targets were enriched in kinases, other cytosolic proteins, and transcription factors. In contrast, Rf-associated targets were predominantly classified as family A G protein-coupled receptors, followed by transcription factors and enzymes. Simultaneously, 1577 anti-thrombotic-related genes were retrieved from the GeneCards database. Overall, eight overlapping genes (STAT3, PTAFR, VEGFA, FGF2, HPSE, IL2, HSP90AA1, and LGALS3) were identified by intersecting the compound-associated genes with the anti-thrombotic-related dataset (Figure 10A,B and Figure S1).

### 3.9. Functional Enrichment and Network Analysis of the Eight Shared Genes Associated with Thrombosis

**Figure 11:**
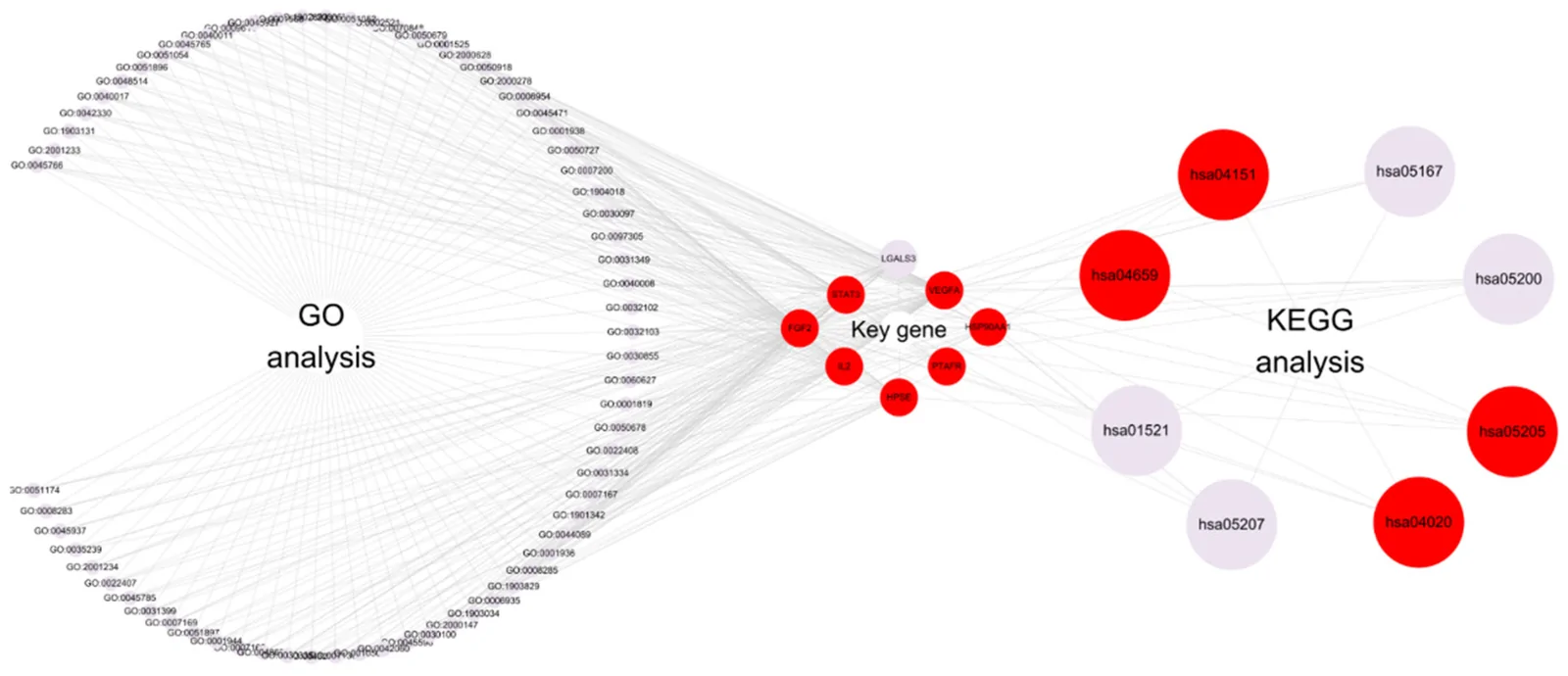
Network pharmacology analysis of white-ginseng-derived ginsenosides related to anti-thrombotic activity: GO–key gene–KEGG network of the eight shared genes. The network was constructed using Cytoscape based on the GO and KEGG enrichment results from Metascape. The central red nodes represent the eight shared genes (STAT3, PTAFR, VEGFA, FGF2, HPSE, IL2, HSP90AA1, and LGALS3). The left side shows enriched GO biological process terms, and the right side shows enriched KEGG pathways. Red KEGG pathway nodes indicate the representative predicted pathways prioritized in this study, including the PI3K–Akt signaling pathway, Th17 cell differentiation, proteoglycan/ECM-related signaling, and the calcium signaling pathway. Light purple KEGG nodes indicate other enriched pathways. Edges indicate gene–term or gene–pathway associations.

Functional enrichment analysis of the eight shared genes using Metascape showed that they were mainly associated with interleukin- and cytokine-related immune signaling, angiogenesis, vasculature development, cell migration, extracellular matrix regulation, and calcium signaling. KEGG pathway analysis further highlighted PI3K–Akt signaling, Th17 cell differentiation, proteoglycan/ECM-related signaling, and calcium signaling as major enriched pathways. These results suggest that the eight shared genes may be associated with inflammatory, vascular, and extracellular matrix-regulatory processes that may be related to thrombosis-related biological pathways (Figure 10C,D). The GO–key gene–KEGG network constructed using Cytoscape showed that the eight shared genes were positioned between the enriched GO terms and KEGG pathways, indicating their functional connectivity with both biological processes and signaling pathways (Figure 11). Among the KEGG pathways, the PI3K–Akt signaling pathway, calcium signaling pathway, Th17 cell differentiation, and proteoglycan/ECM-related signaling were selected as representative predicted pathways and highlighted together with the key genes in the network. These pathways were prioritized because they were more directly associated with the anti-thrombotic and metabolic phenotypes observed in the present study, including lipid metabolism, vascular response, inflammatory signaling, and extracellular matrix remodeling. Although other enriched pathways, such as pathways in cancer, chemical carcinogenesis–receptor activation, EGFR tyrosine kinase inhibitor resistance, and Kaposi sarcoma-associated herpesvirus infection, were also identified, they were not prioritized because the main focus of the present study was the interpretation of anti-thrombotic and metabolic pathway-level hypotheses rather than tumor- or virus-related biology. Although the KEGG term “proteoglycans in cancer” contains a cancer-related label, this pathway was interpreted cautiously as proteoglycan/ECM-related signaling because several predicted genes in this pathway are associated with extracellular matrix regulation, angiogenesis, and vascular microenvironment remodeling. Further pathway mapping using SRplot showed that the shared genes were distributed within these four representative KEGG pathways, suggesting their possible association with inflammatory signaling, vascular regulation, extracellular matrix remodeling, and platelet/vascular response-related processes. However, these results remain computational predictions and require experimental validation (Figures S2–S5).

### 3.10. Molecular Docking Analysis

**Figure 12:**
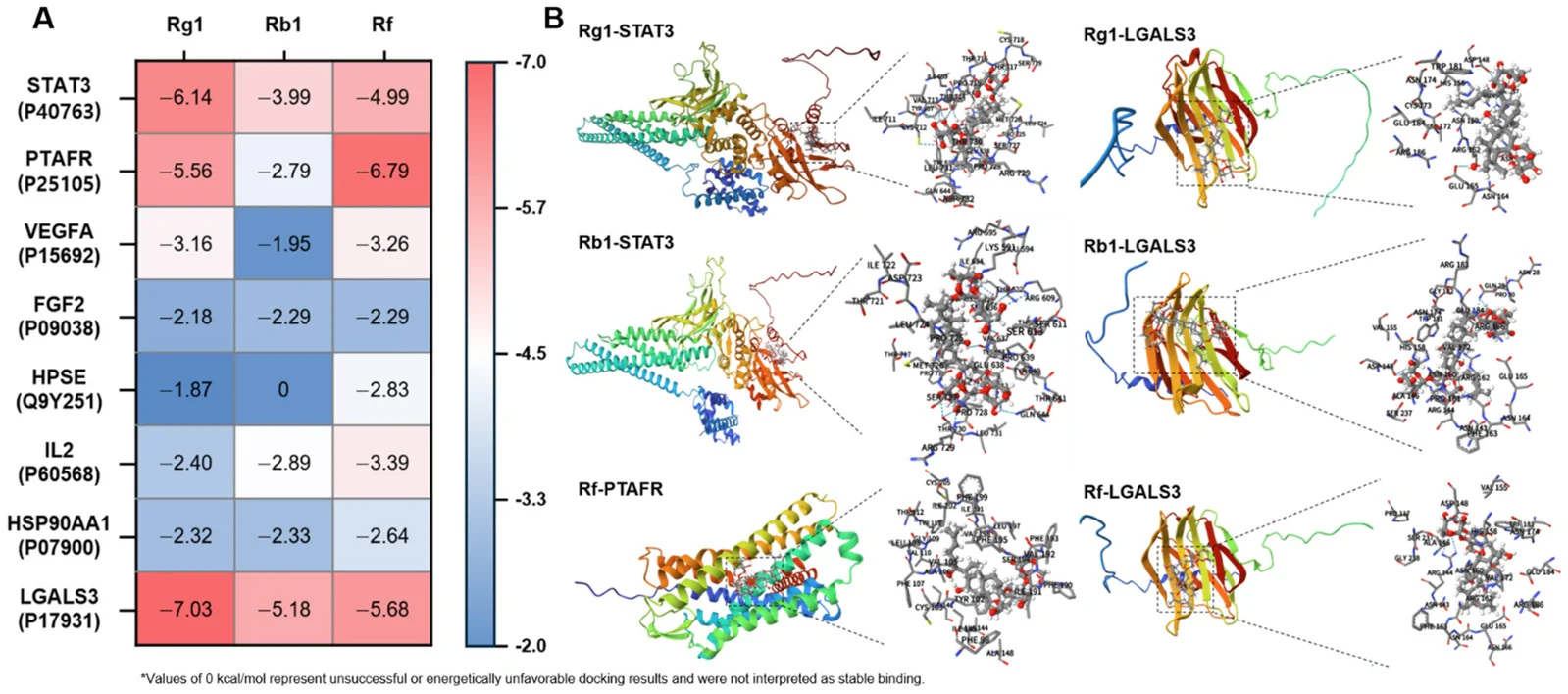
Molecular docking analysis of marker ginsenosides against the eight overlapping target proteins (STAT3, PTAFR, VEGFA, FGF2, HPSE, IL2, HSP90AA1, and LGALS3). (**A**) Heatmap showing the docking affinity values between each single compound and target protein. Stronger predicted binding affinity is indicated in red. (**B**) Representative docking poses of the two highest affinity interactions selected for each single compound. Values marked as 0* represent unsuccessful or energetically unfavorable docking results and were not interpreted as stable binding. Abbreviations: STAT3, signal transducer and activator of transcription 3; PTAFR, platelet-activating factor receptor; VEGFA, vascular endothelial growth factor A; FGF2, fibroblast growth factor 2; HPSE, heparanase; IL2, interleukin-2; HSP90AA1, heat shock protein 90 alpha family class A member 1; LGALS3, galectin-3.

Molecular docking analysis was performed to assess the binding affinities of the three marker ginsenosides against the selected hub proteins. Protein structures were obtained through the AD3.io platform, and AlphaFold-based structural prediction was applied according to the method reported by Jumper et al. [39]. Some compound–protein pairs yielded abnormally large positive docking scores in the AD3.io platform. These results were interpreted as unsuccessful or energetically unfavorable docking events, possibly due to failure to generate a stable binding pose, steric incompatibility, insufficient favorable interactions, or unfavorable structural contacts. Therefore, these pairs were excluded from the interpretation of favorable compound–target interactions. Overall, the docking results were used only as supportive computational evidence for prioritizing putative compound–target interactions, and further experimental validation is required. The docking scores varied depending on the compound–protein pairs, ranging from 0 to −7.03 kcal/mol. In this analysis, a value of 0 was assigned when a positive docking score was obtained, indicating that stable docking was not achieved for that interaction. Among the three compounds, Rg1 showed the strongest overall binding tendency, followed by Rf and Rb1 (Figure 12A). Notably, the strongest interaction for Rg1 was observed with LGALS3 (−7.03 kcal/mol), followed by STAT3 (−6.14 kcal/mol). For Rf, the highest affinity was observed with PTAFR (−6.79 kcal/mol), followed by LGALS3 (−5.68 kcal/mol). In the case of Rb1, the strongest binding was also observed with LGALS3 (−5.18 kcal/mol), followed by STAT3 (−3.99 kcal/mol) (Figure 12B). For each compound, the two interactions with the highest affinity values were selected for further structural comparison. These results suggest that the marker ginsenosides may interact with hub proteins involved in anti-thrombotic-related pathways.

## 4. Discussion

This study aimed to evaluate the anti-thrombotic effect of KGF, obtained through efficient extraction. The contents of the marker compounds, ginsenoside Rg1, Rb1, and Rf, in KGF were quantified using HPLC analysis. The total content of the three ginsenosides present in KGF was determined to be 36.97 mg/g, indicating that this extract possesses a distinct ginsenoside profile compared to conventional white or red ginseng preparations [40,41]. Furthermore, the presence of ginsenoside Rf enables clear differentiation from *Panax quinquefolius*, a species taxonomically distinct from Korean ginseng (*P. ginseng*). Therefore, ginsenosides Rg1, Rb1, and Rf may be considered internationally applicable marker compounds for the quality control of KGF.

To assess the effect, KGF was administered to an HFD model, and its impact on relevant parameters was examined. Rodents fed with a long-term HFD develop metabolic disorders including hyperglycemia, insulin resistance, hepatic steatosis, hyperlipidemia, and obesity-mediated low-grade chronic inflammation [10,13,42]. As expected, in this study, there was a significant increase in body weight, fat deposition, and adipocyte hypertrophy in the rats fed the HFD for 10 weeks. They also had elevated serum levels of total cholesterol, triglycerides, and LDL. The serum levels of HDL, AST, and ALT did not change, as well as the exhibited liver steatosis.

Recent studies have suggested that ginseng may have an anti-obesity effect by reducing the intestinal absorption capacity of dietary fat, regulating the hypothalamic expression of orexigenic neuropeptide Y, or by facilitating the activity of lipoprotein lipase [42,43,44]. Although the HFD group exhibited greater body weight gain than the CONT group, no significant differences in body weight gain were observed among the KGF-treated and HFD groups during the 6-week treatment period. Average daily food intake did not differ significantly among the experimental groups. Because food intake did not differ significantly among the groups, the reduced adipocyte size observed after KGF treatment may reflect altered lipid metabolism rather than reduced caloric intake. In addition, KGF intake significantly reduced the size of epididymal adipocytes caused by fat accumulation. Interestingly, serum leptin levels were significantly reduced in the KGF groups, but not at the highest dose. Although this observation suggests a non-monotonic dose–response relationship, the underlying biological mechanisms remain unclear. Therefore, any explanation for this response should be considered speculative until confirmed by further mechanistic studies. Notably, despite the absence of a significant reduction in leptin levels, the KGF200 group showed improvements in several other metabolic parameters, including serum lipid profiles and hepatic steatosis.

The efficacy of hypolipidemic agents is generally assessed on the basis of reductions in serum total cholesterol, triglycerides, and LDL, and increases in HDL levels [45,46]. In our results, hyperlipidemia induced by a high-fat diet was significantly inhibited by KGF 200 treatment, as evidenced by a reduction in serum total cholesterol and triglycerides.

In general, hepatocyte hypertrophy and ectopic accumulation of fat in the liver (steatosis) are accompanied with increased serum levels of both AST and ALT in both rodent HFD experiments and human clinical meta-analyses [47,48]. A previous study in a Chinese population reported that non-alcoholic fatty liver disease was associated with changes in renal function markers, including serum BUN and creatinine [42]. However, in the present study, serum ALT, AST, BUN, and creatinine levels did not differ significantly among the experimental groups. Thus, the liver and kidney biochemical markers evaluated in this study did not follow the patterns reported in some previous studies. Despite the absence of significant changes in these serum biochemical markers, histological analysis demonstrated that KGF200 attenuated hepatic steatosis, with reduced lipid vacuolation under the present experimental conditions.

In general, orally administered ginseng is exposed to gastric juices, digestive enzymes, and gut microbiota in the gastrointestinal tract, where it is metabolized into a variety of active compounds, including ginsenosides. The extent to which ginseng is broken down and absorbed into its active constituents varies depending on an individual’s digestive capacity and gut microbial composition. Among these metabolites, certain ginsenoside derivatives, such as Compound K, have been reported to improve adipocyte hypertrophy and lipid metabolism. In the present study, the administration of white ginseng (KGF) improved several HFD-associated metabolic parameters, which may partly be related to the contribution of bioactive constituents or metabolites generated after oral administration. Therefore, these findings suggest that oral intake of KGF may contribute to the improvement of lipid metabolism under HFD conditions, although individual variations in metabolic response and the contribution of unmeasured constituents should be considered. Because KGF is a complex ginseng extract, Rg1, Rb1, and Rf should be regarded as marker compounds for quality control and computational analysis rather than as compounds representing the full chemical or pharmacological profile of KGF.

Ginseng is known to be effective in improving blood flow and preventing cardiovascular dysfunction, and has been reported to contribute to vascular health [30]. In the present study, KGF improved obesity-associated metabolic disturbances and exhibited beneficial effects on blood flow in the HFD-induced obesity model.

Ginseng saponins, including ginsenosides, are the principal active constituents of ginseng and are known to exert various pharmacological effects, such as cardioprotective and hepatoprotective activities [24]. It has also been suggested that ginsenosides may act as key components influencing physiological hemostatic and pathological thrombotic processes [49]. Several researchers have reported on the anti-platelet activity of ginseng. Among the active components of ginseng, ginsenosides are considered major bioactive compounds and have been reported to contribute to its metabolic and anti-inflammatory effects [50]. To explore potential anti-thrombotic efficacy, a ferric chloride-induced carotid artery thrombosis animal model was used. When ferric chloride is applied to the tunica adventitia of a blood vessel, it causes oxidative stress in the blood vessels, and the endothelial cells lose their ability to prevent the activation of circulating platelets and the coagulation cascade [51]. This model has been widely used to validate the efficacy of anti-thrombotic agents because of the simplicity of the experimental procedures and the relatively high sensitivity of the results [52]. Importantly, the ferric chloride-induced thrombosis model used in the present study is closely associated with oxidative vascular injury. Previous studies have demonstrated that ferric chloride promotes the generation of reactive oxygen species and oxidative endothelial damage, leading to platelet activation and thrombus formation [53,54]. As shown in Figure 8, KGF treatment tended to delay the reduction in carotid blood flow following FeCl_3_ exposure in a dose-dependent manner. The KGF200 group exhibited a blood flow pattern similar to that observed in the aspirin-treated group. Because this experiment was performed using a limited number of animals (n = 2 per group), these findings should be considered preliminary. Accordingly, the protective effects of KGF observed in this model may be associated with multiple biological activities of ginseng-derived constituents, although the involvement of oxidative stress-related mechanisms was not directly evaluated in the present study. Taken together, these preliminary findings suggest that KGF may have potential anti-thrombotic and anti-platelet effects in vivo.

**Figure 13:**
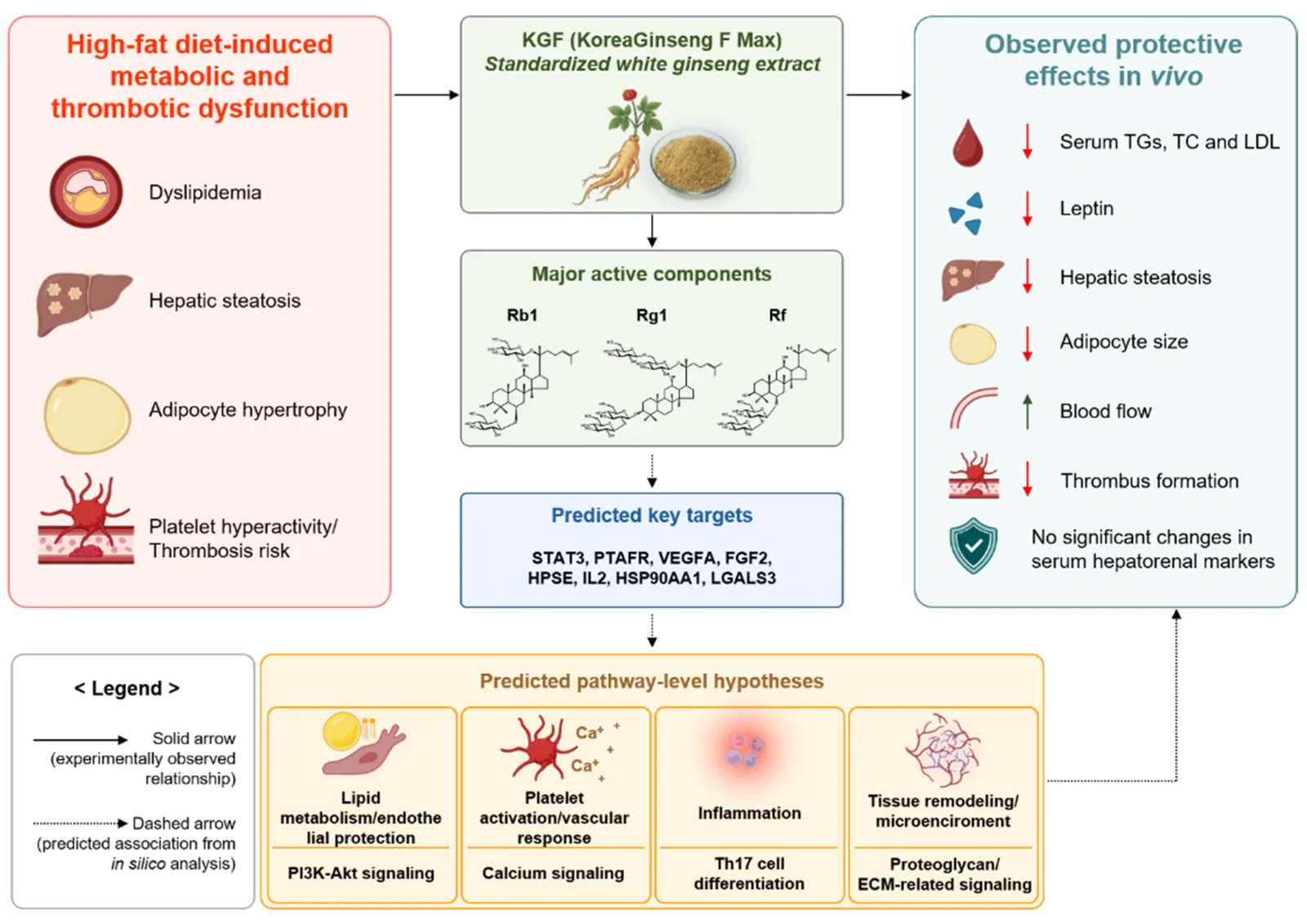
Proposed mechanistic hypothesis of KGF against HFD-induced metabolic and thrombotic dysfunction based on in vivo findings and complementary in silico predictions.

To further generate mechanistic hypotheses based on the in vivo findings, a complementary network pharmacology analysis was performed using the eight overlapping genes identified between the marker compounds and the anti-thrombotic-related dataset. The GO/KEGG network and pathway mapping indicated that these shared genes were mainly associated with inflammatory signaling, vascular regulation, extracellular matrix remodeling, and platelet/vascular response. Because the GO/KEGG enrichment analysis was based on only eight shared genes, the pathway results may be sensitive to individual genes and should be considered exploratory pathway-level predictions rather than robust mechanistic evidence. In particular, PI3K–Akt signaling may represent a potential vascular regulatory pathway, consistent with previous reports showing that ginsenosides Rb1 and Rg1 exert endothelial and cardiovascular protective effects through PI3K/Akt-related mechanisms [55,56]. Calcium signaling and PTAFR were identified through the in silico analysis as potential factors associated with platelet and vascular responses. Consistent with this broader interpretation, Rg1 and Rb1 have been reported to inhibit platelet activation and arterial thrombosis [57,58]. Th17 cell differentiation may represent a potential inflammatory pathway related to obesity-associated low-grade inflammation, as ginsenosides Rg1 and Rb1 have been reported to suppress Th17-related immune responses [59]. Although the KEGG term “proteoglycans in cancer” contains a cancer-related label, this pathway was not interpreted here in the context of tumor biology. Instead, this pathway was interpreted as proteoglycan- and ECM-related signaling rather than tumor-specific signaling because HPSE is involved in ECM remodeling [60], while LGALS3 is associated with VEGFA-dependent endothelial signaling [61]. In addition, the predicted target genes highlighted in the network, including STAT3, PTAFR, VEGFA, FGF2, HPSE, IL2, HSP90AA1, and LGALS3, suggest that KGF may be associated with multiple inflammation-, vascular-, and ECM-related molecular targets. IL2 may be linked to Th17-related inflammatory signaling, and HSP90AA1 to stress-response/chaperone regulation; however, both remain computational predictions requiring experimental validation. Molecular docking analysis was then used as supporting computational evidence to prioritize potential interactions between the marker ginsenosides and these predicted targets, with Rg1 showing the strongest overall binding tendency, followed by Rf and Rb1. Overall, the docking results should be interpreted only as supportive computational evidence for prioritizing putative compound–target interactions, and further experimental validation is required (Figure 13).

Overall, this study demonstrated that KGF treatment showed potential beneficial effects on FeCl_3_-induced thrombotic responses in a high-fat diet-induced obese rat model. Among the tested doses, KGF200 showed the most consistent overall improvements across multiple metabolic and thrombotic parameters. These findings suggest that KGF treatment may have potential as a natural agent for improving metabolic dysfunction and thrombotic risk associated with obesity. However, further studies are needed to elucidate the molecular pathways potentially associated with these effects and to confirm the efficacy and safety of KGF 200 through clinical trials in humans.

In addition, the in silico analysis was limited to the marker compounds Rg1, Rb1, and Rf, although KGF may contain other bioactive constituents and potentially active in vivo metabolites. One of the major limitations of the present study is that oxidative-stress-related biomarkers were not directly measured despite the use of a FeCl_3_-induced oxidative injury model. Therefore, the involvement of antioxidant mechanisms in the observed protective and anti-thrombotic effects of KGF remains to be established. Another limitation of the present study is that only male rats were included. Therefore, the potential influence of biological sex on the therapeutic effects of KGF could not be evaluated, and the generalizability of the present findings remains limited.

Further studies evaluating ROS production, lipid peroxidation, and endogenous antioxidant defense systems, larger sample sizes for thrombosis experiments, and both male and female animals will be necessary to further clarify the underlying mechanisms and therapeutic potential of KGF.

## Figures and Tables

**Table 1:** The concentration data for ginsenosides Rg1, Rf, and Rb1 analyzed by HPLC-UV.

Analyte	Regression Equation (Y = aX + b)	Correlation Coefficient (*R*^2^)	LOD^1)^ (μg/mL)	LOQ^2)^ (μg/mL)	Concentrations (mg/g)
Ginsenoside Rg1	Y = 33.1753X + 20.483	0.9998	2.328	7.977	7.03 ± 0.03
Ginsenoside Rf	Y = 25.2147X + 70.4378	0.9992	1.342	4.401	3.35 ± 0.01
Ginsenoside Rb1	Y = 37.3937X + 130.6781	0.9983	1.11	4.11	26.58 ± 0.19

## Data Availability

All data generated or analyzed during this study are included in the published article and its Supplementary Information files.

## Supplementary Materials

The following supporting information can be downloaded at: https://www.mdpi.com/article/10.3390/antiox15080931/s1, Figure S1: Target class distribution of the predicted targets of ginsenoside Rg1, Rb1, and Rf; Figure S2: KEGG pathway maps of the eight overlapping genes. Green boxes indicate genes mapped to each pathway; Figure S3: KEGG pathway maps of the eight overlapping genes. Green boxes indicate genes mapped to each pathway; Figure S4: KEGG pathway maps of the eight overlapping genes. Green boxes indicate genes mapped to each pathway; Figure S5: KEGG pathway maps of the eight overlapping genes. Green boxes indicate genes mapped to each pathway; Table S1: Effects of KGF in the weight parameters of rats.; Table S2: Blood biochemistry of rats between groups.; Table S3: Composition and caloric content of the control and high-fat diets used in this study.

## Abbreviations

The following abbreviations are used in this manuscript:

ACE	Angiotensin-converting enzyme
CD143	Cluster of differentiation 143
AD3	AD3.io platform
Akt	Protein kinase B
ALT	Alanine aminotransferase
ANOVA	Analysis of variance
ASP	Aspirin
AST	Aspartate aminotransferase
BUN	Blood urea nitrogen
BW	Body weight
CCA	Common carotid artery
CONT	Normal control
COX	Cyclooxygenase
CVD	Cardiovascular disease
EAT	Epididymal adipose tissue
ECM	Extracellular matrix
EGFR	Epidermal growth factor receptor
ELISA	Enzyme-linked immunosorbent assay
eNOS	Endothelial nitric oxide synthase
FeCl3	Ferric chloride
FGF2	Fibroblast growth factor 2
GMP	Good Manufacturing Practice
GO	Gene Ontology
H&E	Hematoxylin and eosin
HDL	High-density lipoprotein
HPLC-UV	High-performance liquid chromatography-ultraviolet detection
HPSE	Heparanase
HSP90AA1	Heat shock protein 90 alpha family class A member 1
IL2	Interleukin-2
KEGG	Kyoto Encyclopedia of Genes and Genomes
KGF	KoreaGinseng F Max
LDL	Low-density lipoprotein
LGALS3	Galectin-3
LOD	Limit of detection
LOQ	Limit of quantification
MFDS	Ministry of Food and Drug Safety
NIH	National Institutes of Health
PAI-1	Plasminogen activator inhibitor-1
PDB	Protein Data Bank
PI3K	Phosphatidylinositol 3-kinase
PTAFR	Platelet-activating factor receptor
SD	Standard deviation
SEM	Standard error of the mean
STAT3	Signal transducer and activator of transcription 3
TC	Total cholesterol
TG	Triglyceride
Th17	T helper 17
VEGFA	Vascular endothelial growth factor A
WHO	World Health Organization
