# Supplementary material for: Anti-Thrombotic and Metabolic Protective Effects of Ginseng in High-Fat Diet-Induced Obese Rats: In Vivo Evaluation with In Silico Mechanistic Prediction
Source: Antioxidants (Basel). 2026 Jul 27;15(8):931. doi: 10.3390/antiox15080931 (PMC13509350; doi:10.3390/antiox15080931)
Supplement: Supplementary file 1 [file antioxidants-15-00931-s001.zip › Supplementary Tables.pdf]

Supplementary Table S1. Effects of KGF in the weight parameters of rats.

	CONT	HFD	HFD+KGF50	HFD+ KGF100	HFD+ KGF200	ASP
Original BW (g)	310.3 ± 22.4 (7.47)	347.3 ± 43.1 (14.38)	356.9 ± 17.4 (5.80)	357.6 ± 24.0 (8.0)	351.6 ± 32.3 (10.75)	359.0 ± 13.5 (4.50)
Final BW (g)	426.6 ± 33.8 (11.28)	527.2 ± 49.7 (16.58)	509.9 ± 27.4 (9.15)	503.6 ± 41.8 (13.92)	513.8 ± 39.3 (13.23)	530.7 ± 36.2 (12.07)
BW gain (g)	116.2 ± 41.1 (15.69)	179.9 ± 25.9 (8.64) **	153.0 ± 38.2 (12.73)**	146.0 ± 29.7 (9.91)	162.2 ± 25.1 (8.38)	171.7 ± 32.2 (10.74)*
Liver (g)	11.17 ± 1.74 (0.58)	11.57 ± 1.99 (0.66)	12.33 ± 2.13 (0.71)	12.66 ± 1.67 (0.56)	11.30 ± 1.33 (0.44)	12.21 ± 1.45 (0.48)
Liver/BW (%)	2.68 ± 0.26 (0.09)	2.32 ± 0.35 (0.12)	2.52 ± 0.35 (0.12)	2.64 ± 0.39 (0.13)	2.32 ± 0.27 (0.09)	2.42 ± 0.32 (0.11)
EAT (g)	1.91 ± 0.31 (0.10)	2.48 ± 0.68 (0.23)	2.39 ± 0.72 (0.24)	2.58 ± 0.25 (0.08)	2.73 ± 0.77 (0.26)	2.30 ± 0.62 (0.21)
EAT/BW (%)	0.46 ± 0.08 (0.03)	0.49 ± 0.10 (0.03)	0.49 ± 0.15 (0.05)	0.54 ± 0.08 (0.03)	0.56 ± 0.15 (0.05)	0.46 ± 0.1 (0.04)

Values were expressed Mean ± SD (SEM). of nine rats (Significant as compared with CONT rats, *P<0.05 and **P<0.01. BW, Body weight, EAT, epididymal adipose tissue).

Supplementary Table S2. Blood biochemistry of rats between groups.

	CONT	HFD	HFD+ KGF50	HFD+ KGF100	HFD+ KGF200	ASP
ALT (U/L)	30.34 ± 4.68	37.70 ± 4.20	37.97 ± 2.55	31.11 ± 2.59	36.97 ± 3.88	29.74 ± 1.97
AST (U/L)	109.74 ± 28.34	107.93 ± 7.55	84.96 ± 8.02	83.20 ± 9.60	95.30 ± 6.15	85.24 ± 3.48
BUN (mg/dL)	20.37 ± 1.04	21.67 ± 1.08	20.51 ± 0.93	22.29 ± 1.76	18.97 ± 1.63	19.74 ± 1.72
Creatinine (mg/dL)	0.47 ± 0.01	0.53 ± 0.05	0.44 ± 0.01	0.55 ± 0.03	0.55 ± 0.09	0.55 ± 0.01
T-chol (mg/dL)	66.57 ± 4.67	108.43 ± 11.64*	92.14 ± 4.45*	85.14 ± 4.78	72.57 ± 6.21$^{\#\#}$	84.29 ± 10.49
TG (mg/dL)	35.71 ± 5.34	66.71 ± 8.13**	46.43 ± 9.31$^{*\#}$	56.86 ± 3.71*	40.00 ± 1.85$^{\#\#}$	38.86 ± 5.39 $^{\#\#}$
HDL (mg/dL)	20.17 ± 1.05	19.17 ± 2.00	24.84 ± 1.32	22.23 ± 1.21	22.79 ± 1.40	24.67 ± 2.45
LDL (mg/dL)	6.64 ± 0.55	12.30 ± 1.77*	12.33 ± 2.72*	11.09 ± 1.61$^{\#}$	7.86 ± 1.05$^{\#\#}$	9.06 ± 0.99$^{\#}$
adiponectin (ng/mL)	3.23 ± 0.29	4.41 ± 0.35	3.82 ± 0.66	3.12 ± 0.22	4.06 ± 0.45	3.50 ± 0.14
Leptin (ng/mL)	1.34 ± 0.32	5.13 ± 0.58***	2.78 ± 0.31$^{\#}$	2.47 ± 0.39$^{\#\#}$	4.53 ± 0.63	3.66 ± 0.59
ACE/CD143 (ng/mL)	19.10 ± 1.93	22.12 ± 2.41	20.27 ± 1.61	18.95 ± 2.54	21.11 ± 2.09	22.71 ± 3.72

Values were expressed Mean ± SD of nine rats (Significant as compared with CONT rats, *P<0.05 and **P<0.01; significant as compared with HFD-fed rats; #P<0.05 and $^{\#\#}$P<0.01. ALT, alanine aminotransferase, AST, aspartate aminotransferase, BUN, blood urea nitrogen, creatinine, creatinine, T-chol, total cholesterol, TG, tryglycerides, HDL, high-density lipoprotein, LDL, low density lipoprotein, ACE, angiotensin-converting enzyme (cluster of differentiation 143)

Supplementary Table S3. Composition and caloric content of the control and high-fat diets used in this study.

Component	Control diet	High-fat diet
Protein (%)	22	26.2
Fat (%)	4.5	34.9
Carbohydrate (%)	49.5	26.3
Energy (kcal/g)	3.3	5.24
% Kcal from fat	12.4	60
Manufacturer	Samtako Bio Korea Co., Ltd., Osan, Korea	Research Diets, Inc., New Brunswick, NJ, USA

Supplementary Table S3. Composition and caloric content of the control and high-fat diets used in the study. Nutritional composition and energy density were obtained from the manufacturer's specifications.
